# The nuclear *18S* ribosomal DNAs of avian haemosporidian parasites

**DOI:** 10.1186/s12936-019-2940-6

**Published:** 2019-09-03

**Authors:** Josef Harl, Tanja Himmel, Gediminas Valkiūnas, Herbert Weissenböck

**Affiliations:** 1 Department of Pathobiology, Institute of Pathology, University of Veterinary Medicine, Veterinaerplatz 1, 1210 Vienna, Austria; 2 Nature Research Centre, Vilnius, Lithuania

**Keywords:** *18S* ribosomal RNA, *Plasmodium*, *Haemoproteus*, *Leucocytozoon*, Birth-and-death evolution, Concerted evolution

## Abstract

**Background:**

*Plasmodium* species feature only four to eight nuclear ribosomal units on different chromosomes, which are assumed to evolve independently according to a birth-and-death model, in which new variants originate by duplication and others are deleted throughout time. Moreover, distinct ribosomal units were shown to be expressed during different developmental stages in the vertebrate and mosquito hosts. Here, the *18S* rDNA sequences of 32 species of avian haemosporidian parasites are reported and compared to those of simian and rodent *Plasmodium* species.

**Methods:**

Almost the entire *18S* rDNAs of avian haemosporidians belonging to the genera *Plasmodium* (7), *Haemoproteus* (9), and *Leucocytozoon* (16) were obtained by PCR, molecular cloning, and sequencing ten clones each. Phylogenetic trees were calculated and sequence patterns were analysed and compared to those of simian and rodent malaria species. A section of the mitochondrial *CytB* was also sequenced.

**Results:**

Sequence patterns in most avian *Plasmodium* species were similar to those in the mammalian parasites with most species featuring two distinct *18S* rDNA sequence clusters. Distinct *18S* variants were also found in *Haemoproteus tartakovskyi* and the three *Leucocytozoon* species, whereas the other species featured sets of similar haplotypes. The *18S* rDNA GC-contents of the *Leucocytozoon toddi* complex and the subgenus *Parahaemoproteus* were extremely high with 49.3% and 44.9%, respectively. The *18S* sequences of several species from all three genera showed chimeric features, thus indicating recombination.

**Conclusion:**

Gene duplication events leading to two diverged main sequence clusters happened independently in at least six out of seven avian *Plasmodium* species, thus supporting evolution according to a birth-and-death model like proposed for the ribosomal units of simian and rodent *Plasmodium* species. Patterns were similar in the *18S* rDNAs of the *Leucocytozoon toddi* complex and *Haemoproteus tartakovskyi*. However, the *18S* rDNAs of the other species seem to evolve in concerted fashion like in most eukaryotes, but the presence of chimeric variants indicates that the ribosomal units rather evolve in a semi-concerted manner. The new data may provide a basis for studies testing whether differential expression of distinct *18S* rDNA also occurs in avian *Plasmodium* species and related haemosporidian parasites.

## Background

### Ribosomal RNAs of eukaryotes

Ribosomal RNAs (rRNAs) form the core part of the ribosomes and are essential for protein synthesis in all living organisms. Due to functional constraints rRNAs are among the most conserved nucleic acids in nature, and at the same time they constitute also the vast majority of RNA molecules in cells, e.g., the rRNA content (dry weight) of *Escherichia coli* cells varies from 20% in the early exponential growth phase to 2% in developed cells [1]. These characteristics render rRNAs suitable targets for various molecular genetic approaches. Ribosomal RNAs and their genomic counterparts, the ribosomal DNAs (rDNAs), are the most common targets in hybridization and screenings assays.

**Fig. 1:**
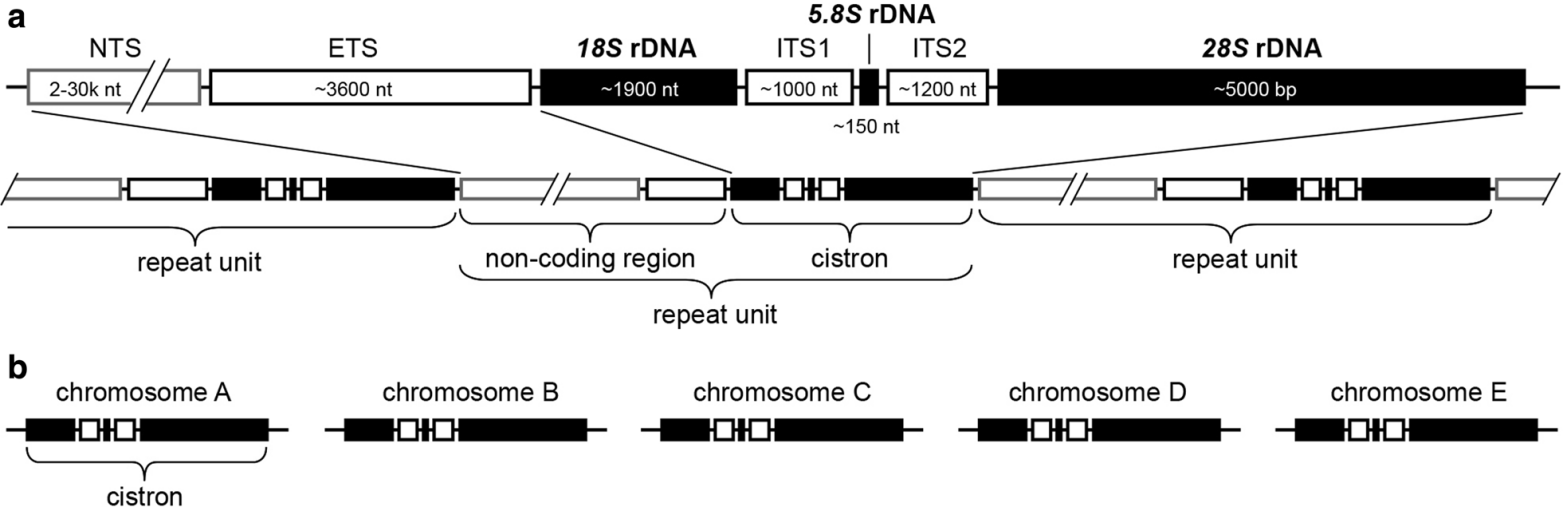
**a** Ribosomal units of multicellular eukaryotes and arrangement in ribosomal clusters (NTS: non-transcribed spacer, ETS: external transcribed spacer). **b** Arrangement of ribosomal units in *Plasmodium* species. The ribosomal units of *Plasmodium* spp. do not contain NTS and ETS regions and are not arranged in clusters with multiple units

Cytoplasmic ribosomes of eukaryotes are composed of four rRNAs and over 50 proteins, which together form the small ribosomal subunit (SSU) and the large ribosomal subunit (LSU). The *18S* rRNA is the rRNA component of the SSU, while *28S* rRNA, *5.8S* rRNA, and *5S* rRNA are the rRNAs of the LSU. Eukaryote nuclear rDNA genes are arranged in units containing *18S* rDNA, *5.8S* rDNA, and *28S* rDNA, separated by the internal transcribed spacers ITS1 and ITS2. The rDNA units of multicellular eukaryotes also include a non-transcribed spacer (NTS) and an external transcribed spacer (ETS), which like ITS1 and ITS2 are not involved in the formation of the ribosomes, but are assumed to play a role in rDNA transcription [2] (Fig. 1a). The *5S* rDNAs are usually not directly associated with the other rDNA units but are located in different genomic regions.

The rDNA units of most eukaryotes are arranged in clusters of so-called tandem repeats on one or several chromosomes, with each cluster containing up to several hundred copies. The individual rDNA units do not evolve independently, but according to a model of concerted evolution, a process that leads to homogenization [3–5]. Concerted evolution involves mechanisms such as unequal crossing over during recombination, gene duplication, and inter-chromosomal gene conversion, and is assumed to affect the evolution of members of most multigene families [6]. However, there are exceptions to this rule. Multigene families involved in the immune system such as immunoglobulins and the major histocompatibility complex (MHC) do not evolve in a concerted fashion [7], but they are assumed to follow a model of birth-and-death evolution [7, 8]. In this model, new copies originate by gene duplication, whereas others become non-functional and deleted over time. In respect to the evolution of their rDNAs, human, simian and rodent *Plasmodium* species are considered exceptional within the eukaryote domain because their rDNA units are assumed to evolve according to a birth-and-death model under strong purifying selection. *Plasmodium* species feature only four to eight rDNA units per haploid genome, which are located on different chromosomes and are assumed to accumulate mutations independently [e.g., 9] (Fig. 1b). Moreover, distinct units were shown to be expressed during different developmental stages of the parasites in the vertebrate and mosquito hosts [10].

### The *18S* rRNAs of *Plasmodium*

The first notable studies on *18S* rDNAs of *Plasmodium* were published in the early 1980s when DNA sequencing was still at the very beginning. Although complete sequences were not available at that time, mapping of rDNAs by restriction enzyme analysis and Southern blot hybridization allowed determining the number of distinct units. By using these methods, four nuclear rDNA units were identified in the rodent malaria parasite *Plasmodium berghei* in different genomic regions, and they were found not to be arranged in tandem arrays like in other eukaryotes [11]. Moreover, the four units could be assigned to two classes, putatively being expressed during different developmental stages [11, 12]. The so-called A-type rDNA units were found to be expressed in asexual blood-stages in the vertebrate host, whereas the expression patterns of the C-type units were still unknown at that time [13]. The naming of rDNA types is quite inconsistent because expression patterns of the C-type rDNAs were unknown at first. Later, McCutchan et al. [14] proposed consistently using the term S-type for the sporozoite specific stages instead of C-type. This recommendation is followed here by using the terms A-type or S-type in the figures and discussion. The first *18S* rDNA sequences of *P. berghei* were published several years later, allowing to directly compare the variants for the first time, and to design oligonucleotide probes specifically targeting the A-type or the C-type [S-type] genes. Hybridization of oligonucleotide probes to rRNA isolated from different life stages lead to the discovery that the C-type [S-type] is expressed in the sporozoite stage in the mosquito vector [15]. Studies on simian malaria species rendered comparable results as those on *P. berghei*. Restriction mapping of rDNA fragments of the human malaria parasite *Plasmodium falciparum* also revealed the presence of at least four distinct units belonging to two classes of transcription units [16]. Two structurally distinct *18S* rDNA genes were sequenced from *P. falciparum* and only the A-type was found to be expressed in blood-stage parasites [17]. Of the two C-type [S-type] rDNAs only one type (C2) was highly expressed in the sporozoite stages [18]. The switch from A-type to C-type [S-type] gene expression in *P. falciparum* was shown to involve the control of rRNA processing [10]. In *Plasmodium cynomolgi*, a parasite of Old World monkeys, five rDNA units were found to be expressed in tissue and blood stages in the vertebrate host, besides a single one being expressed in sporozoites [19]. A-type and S-type *18S* rDNAs were also sequenced and studied in the human malaria species *Plasmodium vivax* [20–22]. Later a third class of rDNA units, the so-called O-type, was characterized in *P. vivax*, which is expressed in ookinetes and oocysts developing in the mosquito midgut [23]. A further class, mainly expressed in maturing ookinetes and young oocysts, was also identified in the rodent malaria parasite *Plasmodium yoelii*. It was termed D-type and characterized as a subtype of the S-type genes [24]. The number of *18S* rDNAs was also determined for the avian parasite *Plasmodium lophurae* using Southern blot analysis and restriction analysis, supporting the presence of six copies, which may be divided into four classes [25].

The temperature was shown to be a major factor involved in transcription of distinct rDNA types. In laboratory cultures of *P. falciparum*, both A-type genes were preferentially expressed at higher temperatures and the S-type genes at lower temperatures. This pattern might relate to differences in body temperatures between the vertebrate and mosquito hosts [18]. Since parasites entering the mosquito host face a severe drop in glucose concentration compared to the vertebrate host, glucose is another potential regulator of gene transcription. Asexual blood stages of *P. falciparum* subjected to glucose starvation decreased expression of A-type genes and increased expression of the S-type genes. This effect was shown to be in synergy with temperature changes related to the host shift [26].

For phylogenetic approaches *18S* rDNAs expressed in asexual stages were sequenced from a wider range of *Plasmodium* species, including *Plasmodium fragile* from macaques [27] and *Plasmodium malariae* from humans [28], and *Plasmodium lophurae* [29] and *Plasmodium gallinaceum* [27] from poultry. The A-type *18S* rDNAs of the primate malaria species *Plasmodium knowlesi* and *Plasmodium cynomolgi* were sequenced by Waters et al. [30]. The first *18S* rDNAs of lizard *Plasmodium* species were published for *Plasmodium mexicanum* (accession number L11716) and *Plasmodium floridense* (L11717) on GenBank only. In a phylogenetic approach, the A-type and S-type *18S* rDNAs were analysed of several simian *Plasmodium* species related to *P. vivax*: *Plasmodium coatneyi*, *Plasmodium inui*, *Plasmodium hylobati*, *Plasmodium fieldi*, *Plasmodium simiovale*, *P. fragile*, and *P. cynomolgi* [31]. The latter simian malaria species are closely related to each other and all feature several A-type gene variants, but only a single S-type gene. Comparison of *18S* rDNAs strongly indicates that gene duplication events resulting in A-type and S-type genes happened independently at least three times in the genus *Plasmodium*, which is again supporting evolution according to a model of birth-and-death evolution [31].

In the last two decades, new data on rDNAs of *Plasmodium* species became available by sequencing complete nuclear genomes of several simian and rodent malaria species. For the first time, not only the numbers and sequences of rDNA units were determined, but they could be assigned to certain chromosomes as well. The first haemosporidian genome published was that of *P. falciparum* (BioProject: PRJNA148 [32]), followed by those of *P. knowlesi* (PRJNA33651, [33]), *P. vivax* (PRJNA20431, [34]), *P. cynomolgi* (PRJDA49901 [35]), *Plasmodium gaboni* (PRJNA329100 [36]), *Plasmodium reichenowi* (PRJNA329100, [36]), *P. coatneyi* (PRJNA329102 [37]), and the two rodent malaria species *P. berghei* (PRJNA317456 [38]) and *P. yoelii* (PRJNA317465 [38]). Annotated nuclear genomes without related publication are available on GenBank from *P. ovale curtisi* (PRJEB12678) and *P. ovale wallikeri* (PRJEB12679), *P. malariae* (PRJEB14392), *Plasmodium chabaudi* (PRJNA317457) and *Plasmodium relictum* (PRJEB9074), whereas only raw genome assemblies are available from *P. inui* (PRJNA257224), *Plasmodium vinckei vinckei* (PRJNA255250), *Plasmodium vinckei petteri* (PRJNA257099) and *P. fragile* (PRJNA282950). The first haemosporidian genome sequenced of a non-*Plasmodium* species was that of *Haemoproteus* (*Parahaemoproteus*) *tartakovskyi* (PRJNA309868 [39]).

The *18S* rDNAs were the first nucleotide sequences available for simian *Plasmodium* species and therefore chosen as targets for parasite screening and detection approaches. Moreover, they feature a succession of extremely conserved and variable sequence regions, which allows detecting and differentiating both high level-taxa (orders, families, genera) and low-level taxa (species, strains). The first approach for distinguishing the human malaria parasites *P. falciparum*, *P. vivax*, *P. malariae*, and *P. ovale* by hybridizing specific oligonucleotide probes to the parasites’ *18S* rRNAs in processed human blood samples followed by autoradiography was developed by Waters and McCutchan [40]. The first comprehensive PCR assay with species-specific primers targeting the *18S* rDNAs of the four above-mentioned species was developed by Snounou et al. [41]. Several other PCR protocols for the detection of simian *Plasmodium* species were published later [42–45].

The *18S* rDNAs were the first sequences analysed in human, simian and rodent *Plasmodium* species, and they are still the targets of choice in molecular screening approaches. Moreover, ribosomal genes of these *Plasmodium* species are assumed to evolve according to a birth-and-death model and to be differentially expressed in the vertebrate and mosquito hosts, respectively. Despite the strong interest in *18S* rDNAs of human, simian and rodent malaria species, ribosomal genes have been neglected widely in studies on avian *Plasmodium* species and related haemosporidians. For the first time, the *18S* rDNAs of a wider range of avian haemosporidians were analysed, including seven *Plasmodium*, nine *Haemoproteus*, and 16 *Leucocytozoon* species. A central question was whether avian haemosporidians feature sequentially distinct ribosomal units like the simian and rodent malaria species, which would be indicative not only for differential expression of distinct rDNA units but also for evolution according to a birth-and-death model. The new data open the way for practical applications in parasite detection (hybridization and PCR assays) but also may facilitate studies on differential gene expression in haemosporidians other than human, simian and rodent *Plasmodium* species.

## Methods

### Sample collection and preparation

For the present study, blood and tissue samples of birds containing parasites of three haemosporidian genera, *Plasmodium*, *Haemoproteus*, and *Leucocytozoon*, were collected. The *18S* rDNAs were analysed of 17 individual samples containing seven *Plasmodium* species belonging to four subgenera (see Table 1): *Plasmodium* (*Haemamoeba*) *relictum* SGS1 (3 individuals), *Plasmodium* (*Haemamoeba*) *matutinum* LINN1 (2)/AFTRU5 (1), *Plasmodium* (*Huffia*) *elongatum* GRW06 (3)/ERIRUB01 (1), *Plasmodium* (*Novyella*) *vaughani* SYAT05 (2), *Plasmodium* (*Novyella*) *delichoni* COLL6 (1), *Plasmodium* (*Giovannolaia*) *homocircumflexum* COLL4 (1), and *Plasmodium* (*Giovannolaia*) sp. WW12 (2). One sample of *Haemoproteus* (*Haemoproteus*) *columbae* COLIV06 was analysed, besides nine samples belonging to eight species of the subgenus *Parahaemoproteus*: *Haemoproteus* (*Parahaemoproteus*) *balmorali* ROBIN1, *Haemoproteus* (*Parahaemoproteus*) *lanii* RBS06, *Haemoproteus* (*Parahaemoproteus*) *minutus* TURDUS2, *Haemoproteus* (*Parahaemoproteus*) sp. EMCIR01, *Haemoproteus* (*Parahaemoproteus*) *syrnii* CULKIB01, *Haemoproteus* (*Parahaemoproteus*) *tartakovskyi* SISKIN1, *Haemoproteus* (*Parahaemoproteus*) sp. LK03, and *Haemoproteus* (*Parahaemoproteus*) sp. STAL2 (2). One sample each was analysed in 16 *Leucocytozoon* species: *Leucocytozoon* cf. *californicus* CIAE02, *Leucocytozoon* sp. SYCON05, *Leucocytozoon* sp. TUPHI06, *Leucocytozoon* sp. PARUS20, *Leucocytozoon* sp. COCOR18, *Leucocytozoon* sp. STAL05, *Leucocytozoon* sp. BUBO01, *Leucocytozoon* sp. ASOT06, *Leucocytozoon* sp. STAL3, *Leucocytozoon* sp. MILVUS2, *Leucocytozoon* sp. COCOR09, *Leucocytozoon* sp. BT1, *Leucocytozoon* sp. COCOR13, *Leucocytozoon* sp. CIAE03, *Leucocytozoon* sp. ACNI03, and *Leucocytozoon* sp. BUTBUT03. The latter three species belong to the *Leucocytozoon toddi* species complex (including *Leucocytozoon mathisi* and *Leucocytozoon buteonis*), parasites of accipitriform predators, which form a clade distinct from that comprising the majority of other *Leucocytozoon* species. The codes following the species names correspond to lineages assigned in the MalAvi database [46], which serves as a reference collection containing most *CytB* haplotypes isolated from avian haemosporidians. Most samples were collected from birds at the University of Veterinary Medicine Vienna (Austria). Blood spots were taken from living birds at the Bird and Reptile Clinic (Department for Companion Animals and Horses) (19 samples), and tissue samples were obtained from the tissue collections of the Research Institute of Wildlife Ecology (7) and the Institute of Pathology (3). Two samples were collected at the Biological Station Neusiedler See (Illmitz, Burgenland). Blood samples were obtained from seven living birds caught at the Ornithological Station in Ventes Ragas, Lithuania. Three samples were obtained from experimentally infected canaries at the Nature Research Centre in Vilnius (Lithuania). The sample of *H. columbae* was collected from blood of *Columba livia* in Cape Town, South Africa. Blood samples of living birds were taken by puncturing the brachial vein and using heparinized microcapillaries. A drop of blood was transferred to filter papers of which DNA was extracted later. Tissue samples were taken from organs (liver and spleen) of dead birds stored at minus 80 °C. DNA was isolated either from blood spots on filter papers or frozen liver tissue using the DNeasy Blood & Tissue Kit (QIAGEN, Venlo, Netherlands). The manufacturer’s protocol for isolation of DNA from tissue was followed, but two eluates of 100 µl each were made from the same column, the first at 8000 rpm, and the second at 13,000 rpm. The second eluate was used for the PCRs.

**Table 1:** Samples analysed in the present study for the *18S* rDNAs and *CytB*

ID	MalAvi lineage	Species	Host species	Origin	*18S* clones no.	*18S* accession no.	*CytB* accession no.
AH0013P	SGS1	*Plasmodium* (*Haemamoeba*) *relictum*	*Serinus canaria*	LT, Vilnius, Nature Research Centre	11	MK650473–MK650483	MK652231
AH0069P	SGS1	*P.* (*Haemamoeba*) *relictum*	*Parus major*	AT, Vienna, VetMed (Pathology)	11	MK650484–MK650494	MK652232
AH0392P	SGS1	*P.* (*Haemamoeba*) *relictum*	*Athene noctua*	AT, Vienna, VetMed (FIWI)	10	MK650495–MK650504	MK652233
AH0792P	AFTRU5	*P.* (*Haemamoeba*) *matutinum*	*Phoenicurus ochruros*	AT, Vienna, VetMed (Bird clinic)	12	MK650524–MK650535	MK652236
AH0524P	LINN1	*P.* (*Haemamoeba*) *matutinum*	*Turdus merula*	AT, Vienna, VetMed (Bird clinic)	9	MK650515–MK650523	MK652235
AH0079P	LINN1	*P.* (*Haemamoeba*) *matutinum*	*Turdus merula*	AT, Vienna, VetMed (Pathology)	10	MK650505–MK650514	MK652234
AH0846P	GRW06	*P.* (*Huffia*) *elongatum*	*Picus viridis*	AT, Vienna, VetMed (Bird clinic)	9	MK650564–MK650572	MK652240
AH0808P	GRW06	*P.* (*Huffia*) *elongatum*	*Parus major*	AT, Vienna, VetMed (Bird clinic)	10	MK650554–MK650563	MK652239
AH0420P	GRW06	*P.* (*Huffia*) *elongatum*	*Corvus corone*	AT, Vienna, VetMed (FIWI)	8	MK650546–MK650553	MK652238
AH0012P	ERIRUB01	*P.* (*Huffia*) *elongatum*	*Serinus canaria*	LT, Vilnius, Nature Research Centre	10	MK650536–MK650545	MK652237
AH0011P	COLL6	*P.* (*Novyella*) *delichoni*	*Carduelis spinus*	LT, Ventes Ragas, Ornithological Station	10	MK650573–MK650582	MK652241
AH0551P	SYAT05	*P.* (*Novyella*) *vaughani*	*Turdus merula*	AT, Vienna, VetMed (Bird clinic)	11	MK650583–MK650593	MK652242
AH0824P	SYAT05	*P.* (*Novyella*) *vaughani*	*Turdus merula*	AT, Vienna, VetMed (Bird clinic)	6	MK650594–MK650599	MK652243
AH0010P	COLL4	*P.* (*Giovannolaia*) *homocircumflexum*	*Serinus canaria*	LT, Vilnius, Nature Research Centre	12	MK650600–MK650611	MK652244
AH0017P	WW12	*P.* (*Giovannolaia*) sp.	*Carduelis spinus*	LT, Vilnius, Nature Research Centre	11	MK650612–MK650622	MK652245
AH0018P	WW12	*P.* (*Giovannolaia*) sp.	*Carduelis spinus*	LT, Vilnius, Nature Research Centre	9	MK650623–MK650631	MK652246
CL017	COLIV03	*Haemoproteus* (*Haemoproteus*) *columbae*	*Columba livia*	ZA, Cape Town, FitzPatrick Institute	11	MK650632–MK650642	MK652247
AH0004H	ROBIN1	*H.* (*Parahaemoproteus*) *balmorali*	*Luscinia luscinia*	LT, Ventes Ragas, Ornithological Station	10	MK650643–MK650652	MK652248
AH0002H	RBS06	*H.* (*Parahaemoproteus*) *lanii*	*Lanius collurio*	LT, Ventes Ragas, Ornithological Station	10	MK650653–MK650662	MK652249
AH0014H	TURDUS2	*H.* (*Parahaemoproteus*) *minutus*	*Turdus merula*	LT, Ventes Ragas, Ornithological Station	8	MK650663–MK650670	MK652250
AH0775H	CULKIB01	*H.* (*Parahaemoproteus*) *syrnii*	*Strix uralensis*	AT, Vienna, VetMed (Bird clinic)	9	MK650671–MK650679	MK652251
AH0005H	SISKIN1	*H.* (*Parahaemoproteus*) *tartakovskyi*	*Loxia curvirostra*	LT, Vilnius, Nature Research Centre	10	MK650680–MK650689	MK652252
AH0141H	STAL2	*H.* (*Parahaemoproteus*) sp.	*Strix uralensis*	AT, Vienna, VetMed (FIWI)	8	MK650690–MK650697	MK652253
AH0460H	LK03	*H.* (*Parahaemoproteus*) sp.	*Falco tinnunculus*	AT, Vienna, VetMed (Bird clinic)	9	MK650698–MK650706	MK652254
AH0608H	EMCIR01	*H.* (*Parahaemoproteus*) sp.	*Emberiza citrinella*	AT, Vienna, VetMed (Pathology)	6	MK650707–MK650712	MK652255
AH0776H	STAL2	*H.* (*Parahaemoproteus*) sp.	*Strix uralensis*	AT, Vienna, VetMed (Bird clinic)	11	MK650713–MK650723	MK652256
AH0255L	CIAE02	*Leucocytozoon* cf. *californicus*	*Circus aeruginosus*	AT, Vienna, VetMed (FIWI)	12	MK650775–MK650786	MK652257
AH0040L	STAL3	*Leucocytozoon* sp.	*Strix aluco*	AT, Vienna, VetMed (Bird clinic)	10	MK650724–MK650733	MK652258
AH0053L	TUPHI06	*Leucocytozoon* sp.	*Turdus merula*	AT, Vienna, VetMed (Bird clinic)	2	MK650741–MK650742	MK652259
AH0110L	PARUS20	*Leucocytozoon* sp.	*Corvus corone cornix*	AT, Vienna, VetMed (Bird clinic)	10	MK650744–MK650753	MK652260
AH0145L	STAL5	*Leucocytozoon* sp.	*Strix aluco*	AT, Vienna, VetMed (FIWI)	10	MK650754–MK650763	MK652261
AH0232L	MILVUS02	*Leucocytozoon* sp.	*Haliaeetus albicilla*	AT, Vienna, VetMed (FIWI)	11	MK650764–MK650774	MK652262
AH0286L	COCOR09	*Leucocytozoon* sp.	*Corvus corone*	AT, Vienna, VetMed (FIWI)	10	MK650787–MK650796	MK652263
AH0439L	COCOR18	*Leucocytozoon* sp.	*Corvus corone cornix*	AT, Vienna, VetMed (Bird clinic)	2	MK650802–MK650803	MK652264
AH0441L	COCOR13	*Leucocytozoon* sp.	*Corvus corone corone*	AT, Vienna, VetMed (Bird clinic)	11	MK650806–MK650816	MK652265
AH0517L	ASOT06	*Leucocytozoon* sp.	*Asio otus*	AT, Vienna, VetMed (Bird clinic)	10	MK650817–MK650826	MK652266
AH0856L	BT1	*Leucocytozoon* sp.	*Phylloscopus trochilus*	AT, Burgenland, B.S. Illmitz	11	MK650827–MK650837	MK652267
AH0932L	SYCON05	*Leucocytozoon* sp.	*Sylvia atricapilla*	AT, Burgenland, B.S. Illmitz	10	MK650838–MK650847	MK652268
AH0994L	BUBO01	*Leucocytozoon* sp.	*Bubo bubo*	AT, Vienna, VetMed (Bird clinic)	11	MK650848–MK650858	MK652269
AH0555L	BUTBUT03	*Leucocytozoon* sp. (*L. toddi* complex)	*Buteo buteo*	AT, Vienna, VetMed (Bird clinic)	11	MK650859–MK650869	MK652270
AH0799L	ACNI03	*Leucocytozoon* sp. (*L. toddi* complex)	*Accipiter nisus*	AT, Vienna, VetMed (Bird clinic)	10	MK650870–MK650879	MK652271
AH1003L	CIAE03	*Leucocytozoon* sp. (*L. toddi* complex)	*Circus aeruginosus*	AT, Vienna, VetMed (Bird clinic)	12	MK650880–MK650890	MK652272

Indicated are MalAvi lineage names, parasite species, host species, origin of samples, number of clones sequenced, and accession numbers for the *18S* rDNA and *CytB* sequences deposited in NCBI GenBank

### *18S* rDNA primers

Complete *18S* rDNA sequences have been published for several *Plasmodium* species and *H.* (*Parahaemoproteus*) *tartakovskyi*, but no data were available for *Leucocytozoon* spp. and parasites of other genera and subgenera of the order Haemosporida. The *18S* rDNA sequences of *Plasmodium* and *Parahaemoproteus* species differ strongly in sequence composition (genetic distances are high even when conserved sections of the *18S* are compared) and had to be assessed with two separate primer sets. One primer set was designed based on genome sequences of *Plasmodium* spp. and allowed amplifying the *18S* rDNAs of *Plasmodium* spp., *Leucocytozoon* spp., and *Haemoproteus columbae.* Another primer set was based on two *18S* rDNAs isolated from the genome of *Haemoproteus (Parahaemoproteus) tartakovskyi* (PRJNA309868, [39]) and used for *Parahaemoproteus*-positive samples only. To identify sequence regions specific to haemosporidians, and to reduce the possibility of unintentional binding to host DNA, *18S* rDNAs of various bird species were included in the alignment. At first, a nested PCR approach with two forward and reverse primers each was intended, but most samples featured good quality PCR products in the first round, and the second primer sets were used for some samples only. The primers **18S_P_1F** (5′-CAAAGATTAAGCCATGCAAGTGA-3′) and **18S_P_1R** (5′-CGGAAACCTTGTTACGACTTCTC-3′), located about 50 base pairs (bp) from the 5′- and 3′-ends of the *18S* rDNA, were used to amplify the *18S* rDNAs of *Plasmodium* spp., *Leucocytozoon* spp., and *Haemoproteus columbae*. The alternative primers **18S_P_2F** (5′-GAACGGCTCATTAAAACAGTTATAATCT-3′) and **18S_P_2R** (5′-CGACTTCTCCTTCCTTTAAAAGATAG-3′) are shifted inwards by 100 bp and 20 bp, respectively. The primers **18S_H_1F** (5′-TGGTTGATCTTGCCAGTAATATATGT-3′) and **18S_H_1R** (5′-CGGAAACCTTGTTACGACTTTTG-3′), located at the 5′-end and 20 bp from the 3′-end, respectively, were used to amplify the *18S* rDNAs of species belonging to the subgenus *Parahaemoproteus*. The alternative primers **18S_H_2F** (5′-ACGGCTCCTTAAAACCGTTATAATC-3′ and **18S_H_2R** (5′-GCAAAAGGCAGTTACGCATACAG-3′) are shifted inwards by 100 bp and 120 bp, respectively. The primers intended for the nest 1 PCRs allowed for amplification of almost the entire *18S* rDNAs (~ 2100 bp) of the haemosporidians under investigation. Based on the *18S* rDNAs of *Haemoproteus* (*Haemoproteus*) *columbae*, the primers **18S_Hcol_F2** (5′-ACGGCTCCTTAAAACAGTTATAATCT-3′) and **18S_Hcol_F2** (5′-ACGGCTCCTTAAAACAGTTATAATCT-3′) were designed. They were successfully tested on three *H. columbae* lineages (COLIV03, COLIV06, and HAECOL1) but not used in the present publication. The latter primers are provided to facilitate future studies on *18S* rDNAs of the subgenus *Haemoproteus* because neither the **18SL_P** nor the **18SL_H** primer sets allowed for amplification of *18S* rDNAs of other lineages in this group.

### *CytB* primers

From all samples, also a section of the mitochondrial *Cytochrome B* (*CytB*) was sequenced, which is the standard “DNA-barcode” region for the identification of avian haemosporidian lineages. The common nested PCR approach by Hellgren et al. [47] allows obtaining a 478 bp fragment of the *CytB* from most avian haemosporidians. PCRs were performed on all samples using the primers HaemNFI and HaemNR3 in the nest 1 PCR, and HaemF/HaemR2 and HaemFL/HaemR2L in the nest 2 PCRs, respectively. The nest 1 PCRs were performed with the original DNA templates and the nest 2 PCRs with each 1 µl product of the first PCRs as template. However, the primers do not allow amplifying all *CytB* lineages. Samples isolated from birds of the order Accipitriformes were additionally screened with primers specifically targeting parasites of the *L. toddi* species complex, published by Himmel et al. [48]. These primers allow sequencing a 528 bp section of the *CytB*. The primers CytB_L2_F (5′-GAGAGTTATGGGCTGGATGGT-3′) and CytB_L2_R (5′-TAGAAAGCCAAGAAATACCATTCTG-3′) were used in the nest 1 PCRs, and CytB_L2_nF (5′-GCTGGATGGTGTTTTAGATAYATGC-3′) and CytB_L2_nR (5′-CCATTCTGGAACAATATGTAAAGGTG-3′) were used in the nest 2 PCRs and for sequencing, respectively. In addition, general primers were designed, which were used for sequencing an 886 bp *CytB* section in all samples: CytB_HPL_intF1 (5′-GAGAATTATGGAGTGGATGGTG-3′) and CytB_HPL_intR1 (5′-ATGTTTGCTTGGGAGCTGTAATC-3′). The resulting sequences were used for the calculations of the *CytB* trees.

### PCRs and molecular cloning

The PCRs targeting the *18S* rDNAs were performed using the GoTaq^®^ Long PCR Master Mix (Promega, Wisconsin, Madison, USA), which has a proof-reading activity and allows for amplification of long PCR products. Master mixes contained 9.5 µl distilled water, 12.5 µl GoTaq^®^ Long PCR Master Mix 2×, 1 µl of each primer (10 pMol/µl), and 1 µl DNA template. PCRs started with an initial denaturation for 2 min at 94 °C, followed by 35 cycles with 30 s at 94 °C, 30 s at 55 °C, 2 min at 68 °C, and a final extension for 10 min at 72 °C. The PCRs for the 886 bp *CytB* fragment were performed under the same conditions, but with 1 min extension time only. The PCRs targeting all other *CytB* fragments were performed using the GoTaq^®^ G2 Flexi DNA Polymerase (Promega). The PCRs following the protocol of Hellgren et al. [47] started with an initial denaturation for 2 min at 94 °C, followed by 35 cycles with 30 s at 94 °C, 30 s at 50 °C, 1 min at 72 °C, and a final extension for 10 min at 72 °C. Each 1 µl of nest 1 PCR-product was used as template in the nest 2 PCRs. The nested PCRs targeting the *CytB* sequences of the *L. toddi* complex were performed under the same conditions, but at 55 °C in the annealing step. All PCR products were visualized on 1% LB agarose gels using a Gel Doc™ XR + Imager (Biorad, Hercules, California, USA). The *CytB* PCR-products were sent to Microsynth Austria GmbH (Vienna, Austria) for purification and direct sequencing using the PCR primers (nest 2 primers in case of nested PCRs). The *18S* rDNA PCR-products were further processed and subjected to molecular cloning. Each 20 µl of PCR-products were run on 1% LB agarose gels and excised with flamed spatulas. The cut bands were purified using the QIAquick Gel-Extraction Kit (QIAGEN) following the standard protocol and eluted with 20 µl distilled water. Cloning was performed with the TOPO™ TA Cloning™ Kit (Invitrogen, Carlsbad, California, USA) using the pCR™4-TOPO^®^ vector and One Shot^®^ TOP10 competent cells. Following ligation and transformation, *E. coli* cells were recovered in SOC medium for 1 h at 37 °C, and then plated on LB agar plates and grown for 20 h at 37 °C. From each cloning assay, 15 to 20 individual clones were picked with sterilized tooth sticks and transferred to fresh LB agar plates. The same tooth sticks with remaining *E. coli* were twisted in PCR-tubes with 25 µl master mix for the colony-PCRs. Colony-PCRs were performed with the GoTaq^®^ Long PCR Master Mix (Promega) under the same conditions as the *18S* rDNA PCRs (see above), using the primers M13nF (5′-TGTAAAACGACGGCCAGTGA-3′) and M13nR (5′-GACCATGATTACGCCAAGCTC-3′). The PCR-products of clones carrying inserts of the expected size were sent to Microsynth Austria GmbH (Vienna, Austria) for purification and sequencing (using the colony-PCR primers).

### Sequence analyses

The raw forward and reverse sequences (and electropherograms) were carefully checked and aligned in Bioedit v.7.0.8.0 [49]. Following the inspection of individual clones, all sequences were aligned and sorted in MAFFT v.7 [50] applying the default options. In a second control step, all unique positions in the alignment were rechecked in the corresponding electropherograms. For the phylogenetic analyses, GenBank sequences from *Plasmodium* species for which the whole sets of *18S* rDNAs were available (Additional file 1) were included. Sequences of raw genomes were retrieved by performing BLAST searches against the “whole genome shotgun contigs” database of NCBI GenBank. A-type and S-type *18S* rDNAs of several simian *Plasmodium* species published by Nishimoto et al. [31] were also included. The genome of *P. malariae* (PRJEB14392; Wellcome Trust Sanger Institute WTSI, unpublished) features two similar *18S* rDNAs on chromosomes 3 and 10, and an aberrant variant on chromosome 11. The latter variant was too different to be reasonably incorporated in the alignment. Genomic *18S* rDNAs were included of *P. gallinaceum* (PRJEB9073; WTSI, unpublished), but not of the *P. relictum* (PRJEB9074; WTSI, unpublished), because sequences of the latter were fragmentary only. Moreover, the *18S* rDNAs published for *Plasmodium cathemerium* (AY625607, [51]) and *Plasmodium lophurae* (X13706, [29]) were not included because these featured only the A-type sequences. The sequence patterns differed strongly between genera, wherefore separate trees were calculated for *Plasmodium*, *Leucocytozoon*, and *Haemoproteus*. The genetic distances between genera were high and even the most conserved sequence sections differed in multiple nucleotide positions. All datasets were aligned with MAFFT v.7 [50] using the option “E-ins-i” for alignments with multiple conserved domains and long gaps. Uncorrected *p*-distances between *18S* rDNA variants were calculated with MEGA v.7.0.21 [52] based on alignments containing all single haplotypes. The alignments were then refined in Bioedit v.7.0.8.0 [49] and trimmed to the size of the shortest sequence(s). For the phylogenetic tree analyses, a subset of clones from the present data set was selected, which included the main sequence variants isolated from each individual. Prior to the tree calculations, all gap positions were removed using trimAl v.1.2 [53]. The *18S* alignments produced with the latter approach provided the most reasonable result according to visual inspection by the first author, e.g., because identical sequence regions were correctly aligned throughout the alignment, more reliably than by other programs.

Hypervariable regions containing long inserts or deletions were generally removed by deleting all gap positions from the alignment before the analyses. These alignments also resulted in trees featuring the highest support values. Alternatively, for the *Plasmodium* data set only, alignments were also calculated with R-Coffee [54], which uses predicted secondary structures of the ribosomal sequences. Moreover, three different trimming options were applied: (1) only gap positions excluded, (2) “strict” algorithm implemented in trimAl v.1.2 [53], and (3) default options on the G-blocks Server (http://molevol.cmima.csic.es/castresana/Gblocks_server.html) [55]. The GC-contents of sequences were calculated with Microsoft Excel v.2019.

Phylogenetic trees were also calculated with the *CytB* sequences obtained from the same individuals. The *CytB* sequences were of same length (886 bp) and aligned manually. Substitution models were evaluated for all individual data sets using IQ-TREE [56]. According to the corrected Akaike information criterion (cAIC), the optimal substitution model for all alignments was GTR + I + G. Maximum Likelihood (ML) trees were calculated with IQ-TREE [56] by performing 1000 bootstrap replicates each. Bayesian Inference (BI) trees were calculated with MrBayes v.3.2 [57], the analyses were run for 10 million generations each (2 runs each with 4 chains, one of which was heated) and every thousandth tree was sampled. The first 25% of trees were discarded as burn-in and 50% majority rule consensus trees were calculated from the remaining 37,500 trees each. The program RDP4 (Recombination Detection Program v.4; [58, 59]) was used to test whether distinct *18S* variants isolated from the same species showed chimeric features resulting from recombination events. Prior to the RDP4 analyses, the *18S* sequences of each species were separately aligned with MAFFT v.7 [52] using the option “E-ins-i”. Following methods, implemented in RDP4, were used to test for recombination: GENECONV [60], MAXCHI [61], Chimaera [62], SiSscan [63], and 3Seq [64].

## Results

Nuclear *18S* rDNA sequences were obtained from seven avian *Plasmodium* species, nine *Haemoproteus* spp. and 16 *Leucocytozoon* spp. by PCR, cloning and sequencing. Two to twelve clones were sequenced from each sample (404 in total, 9.6 clones in mean; Table 1). The clones of four samples (not listed in publication) were excluded prior to the analyses, because they contained mixed infections with two different *Leucocytozoon* species, which was not visible in the sequences amplified with the primers by [47], but in those obtained with the CytB_HPL_intF1 and CytB_HPL_intR1 primers (present study). Another 39 clones featuring sequences of the bird hosts were also excluded. The *18S* rDNA sequence patterns varied strongly between species, hampering the classification of haplotypes into groups. Haplotypes of some species were clearly distinct from each other, whereas those of others differed only in a few sites. In the following, similar clones or haplotypes are referred to as “variants”, and groups of substantially different variants as “clusters”. Calculations of uncorrected *p*-distances between variants, clusters, and species clades were performed on *18S* rDNA alignments containing all single clones. The alignments for the calculation of phylogenetic trees contained only a selection of *18S* rDNA clones, including the main sequence variants obtained from each specimen. For comparison, already published sequences of simian and rodent malaria species were included in the *Plasmodium* alignment. It must be noted that alignments of ribosomal genes vary quite strongly depending on the taxa included, and variable sections cannot be aligned with confidence between genetically distant taxa and show high levels of saturation. However, variable sections were kept in the alignments by removing only the positions containing gaps, because these contained phylogenetically informative sites on subgroup level. *Plasmodium 18S* rDNA trees calculated based on a secondary structure alignment, applying three different trimming methods, are provided in Additional files S2, S4, and S4. The sequences are deposited at NCBI GenBank under the accession numbers MK650473–MK650890 (*18S* rDNA) and MK652231–MK652272 (*CytB*), respectively.

### *18S* rDNAs of avian *Plasmodium* species

**Fig. 2:**
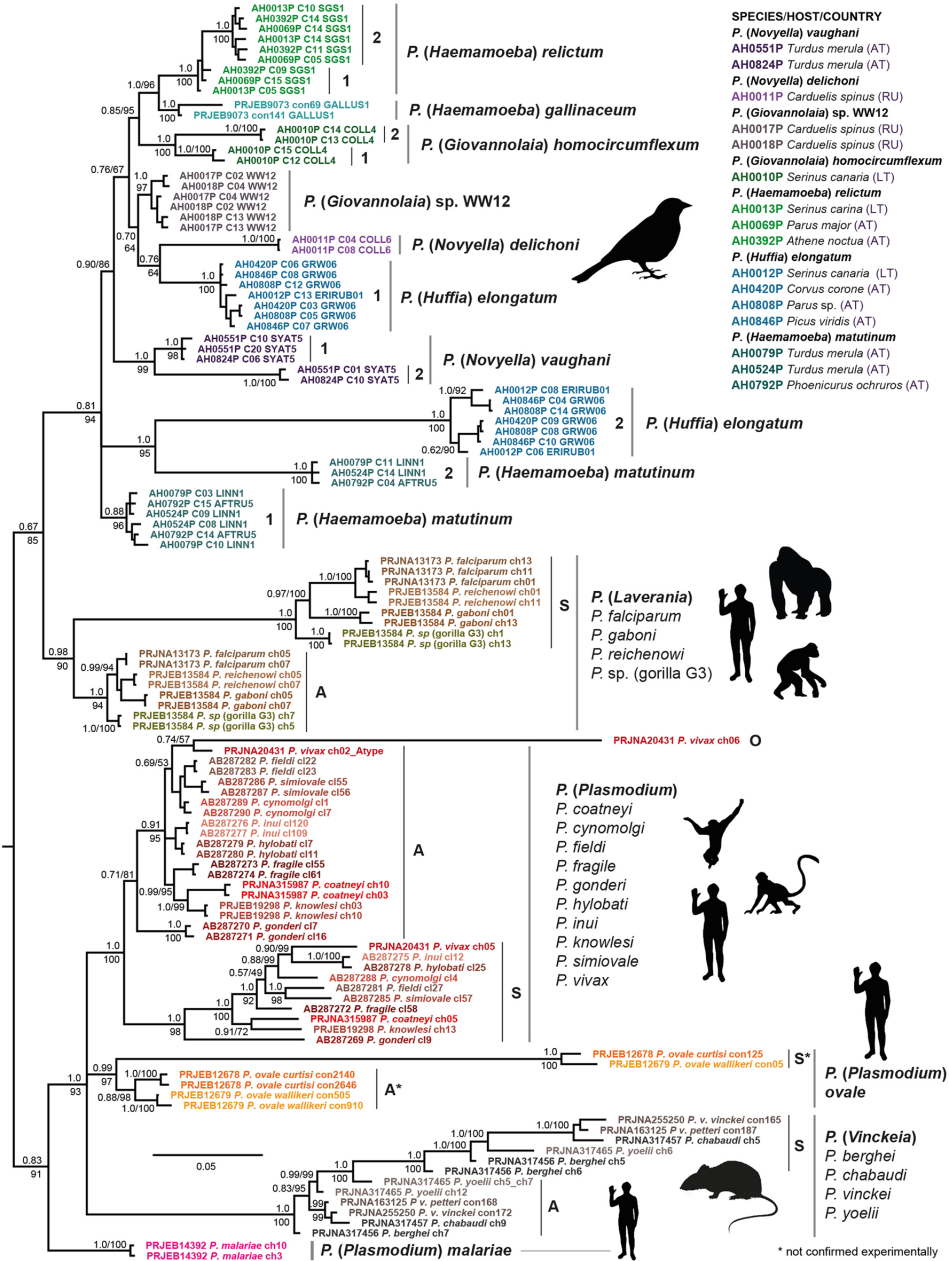
Bayesian inference tree of *Plasmodium 18S* rDNA sequences. Posterior probabilities and maximum likelihood bootstrap values are indicated at most nodes. The scale bar indicates the expected mean number of substitutions per site according to the model of sequence evolution applied. Clades with variants expressed in the vertebrate hosts are labelled with “A”, and clades with sporozoite specific sequences with “S”. As outgroup a sequence of *Leucocytozoon* sp. (MalAvi lineage SYCON05; AH0932L_c07) was included

The *18S* rDNA data set generated in the present study comprised sequences of seven avian *Plasmodium* species (16 specimens; 159 clones, 9.9 mean per sample). Multiple samples were analysed of *Plasmodium elongatum* (4 specimens), *Plasmodium matutinum* (3), *P. relictum* SGS1 (3), *Plasmodium vaughani* (2) and *P.* sp. WW12 (2), and one sample each of *Plasmodium delichoni* and *Plasmodium homocircumflexum*. Conspecifics consistently featured similar sets of sequence variants. Two sequences of the *P. gallinaceum* genome (PRJEB9073, contigs 69 and 141) and 68 sequences from 20 simian and rodent *Plasmodium* species were also included. The *18S* rDNA alignment was trimmed to the length of the shortest sequence (2992 alignment positions) and contained 1654 sites after all positions with gaps were removed. In the *18S* rDNA trees, the avian *Plasmodium* species grouped within a moderately supported clade (BI posterior probability: 0.90/ML bootstrap support: 86) (Fig. 2). Support values at deeper nodes were low and the topology differed partially from the *CytB* trees (Additional file 2). Apart from the alignment and trimming methods used for the main tree shown in Fig. 2, trees were calculated also with the *Plasmodium 18S* sequences aligned with R-Coffee [54], which uses predicted secondary structures of the ribosomal sequences. The tree calculated with the first approach (only gap positions excluded; Additional file 3) shows a similar topology as that presented in Fig. 2, but bootstrap support values are generally lower. Support values were considerably lower in the tree obtained with the second approach (“strict” algorithm implemented in trimAl v.1.2 [53]; Additional file 4). The tree obtained with the third approach, using the most stringent trimming option (default option on the G-blocks Server [55]; Additional file 5), featured the lowest support values, moreover the A-type and S-type clades of the subgenus *Laverania* and *Plasmodium ovale* did not group, respectively.

Distinct *18S* rDNA variants were found in all species, but patterns differed quite strongly between them. The lowest diversity was found in *P. delichoni* (AH0011P) and *Plasmodium* sp. WW12 (AH0017P, AH0018P) with distances of up to 0.6% and 1.6% between different haplotypes, respectively (Additional file 6). *Plasmodium delichoni* featured four similar haplotypes, whereas the *Plasmodium* sp. WW12 samples featured two main variants each, one of which comprised several similar haplotypes little diverged from each other, and the second of which was represented by two clones (AH0017P c2, AH0018P c4) only. *Plasmodium relictum* (AH0013P, AH0069P, AH0392P), *P*. *gallinaceum* (PRJEB9073), *P. homocircumflexum* (AH0010P), and *P. vaughani* (AH0551P, AH0824P) featured two sequence clusters each, whereby different clusters of the same species grouped in all cases. The *P. relictum* clusters differed from each other by 2.3%, with differences mainly in a short section 650 bp to 850 bp from the 5′-end of the *18S* rDNA. The short-branched cluster contained only one or two clones of each sample (AH0013P c5, c7; AH0392 c9; AH0069P c15), the long-branched cluster contained all other haplotypes, being similar to each other, but indicating the presence of three or four additional nuclear *18S* rDNA copies. *Plasmodium gallinaceum* and *P. relictum*, both members of the subgenus *Haemamoeba*, presented as sister lineages in the *18S* rDNA and the *CytB* trees, and featured the same distance between the two genomic copies with 2.3%. The distances between sequence clusters were considerably higher in *P.* (*Giovannolaia*) *homocircumflexum* and *P.* (*Novyella*) *vaughani* with 7.2% and 9.3%, respectively. Both species featured two clusters, which contained at least two distinct sequence variants each, suggesting the presence of four or more nuclear *18S* rDNA copies. Moreover, two clones of *P. homocircumflexum* presented with chimeric features, with sequence sections partially matching variants of one of the two main clusters, respectively. The first half of clone 9 (bp 1 to 900) matched clones 5, 10, 12, 15, and 16, whereas the second half (bp 1050 to 2020) matched clones 2, 6, 11, 13, and 14. The first 1620 bp of clone 3 matched those of clones 5, 10, 12, 15, and 16, but the last section (nt. 1650 to 2020) matched clones 2, 6, 11, 13, and 14. All five recombination tests (GENECONV, MAXCHI, Chimaera, SiSscan, and 3Seq) performed with RDP4 [58, 59] were significant for the presence of recombination signals in the latter two clones (Additional file 7). Highest distances between *18S* rDNA variants were found in *P.* (*Haemamoeba*) *matutinum* (AH0079P, AH0524P, AH0792P) and *P.* (*Huffia*) *elongatum* (AH0012P, AH0420P, AH0808P, AH0846P) with up to 10.8% and 14.9%, respectively. Both species featured two sequence clusters, each containing at least two distinct variants. However, unlike in the other *Plasmodium* species, short-branched and long-branched clusters of the two species did not group in reciprocally monophyletic clades [meaning that all lineages of the same species clustered together into “species” clades]. The long-branched cluster of *P. elongatum* formed a weakly supported clade with *P. delichoni* (0.76/64), whereas that of *P. matutinum* branched off from the basal node of the avian *Plasmodium* clade. The long-branched clusters of *P. elongatum* and *P. matutinum* grouped as sister lineages into one clade (1.0/95), although differing from each other by 16.6%. Moreover, several clones of the long-branched *P. elongatum* cluster presented with chimeric features. Clone 12 of sample AH0012P was largely similar to the clones of the long-branched, but the first 100 bp showed ten mismatches and were identical to sequences of the short-branched cluster, respectively. Other haplotypes of the long-branched cluster showed chimeric features as well, but patterns were complex and not consistent. The RDP4 [58, 59] analyses detected recombinant signals in several *18S* clones of *P. elongatum*, but other than in case of *P. homocircumflexum*, results were significant only in one or two of the tests each (Additional file 7). Recombinant signals were also detected in some of the tests in clones of *P. matutinum* (AH0524P), *P. relictum* (AH0013P, AH0069P, AH0392P) and *P*. sp. WW12 (AH0017P, AH0018P). The GC-content of the *18S* rDNAs ranged from 33.1 to 35.0% (mean 34.0%) and, therefore, was similar in all avian *Plasmodium* species. The approximate total lengths of the avian *Plasmodium 18S* rDNAs (extended by sequence sections missing at the 5′- and 3′-ends) ranged from 2100 to 2177 bp (mean 2138 bp).

### *18S* rDNAs of human, simian and rodent *Plasmodium* species

The *18S* rDNAs of species belonging to subgenus *Laverania* grouped with those of avian haemosporidians with low support (0.81/85). *Plasmodium malariae* formed the sister lineage to a clade containing all other simian and rodent *Plasmodium* species groups except for *Laverania*. This clade presented as a trichotomy with three well supported subclades, the first comprising species of the subgenus *Vinckeia* (*P. berghei*, *P. chabaudi*, *P. berghei*, and *P. yoelii*) (1.0/100), the second *P. ovale wallikeri* and *P. ovale curtisi* (0.99/97), and the third *P. vivax*, *P. coatneyi*, *P. cynomolgi*, *P. fieldi*, *P. fragile*, *P. gonderi*, *P. hylobati*, *P. inui*, *P. knowlesi*, and *P. simiovale* (1.0/100) (Fig. 2). *Plasmodium* species of these clades and the *Laverania* clade featured two sequence clusters with A-type and S-type variants each, indicating that ribosomal variants with differential expression patterns originated independently at least in four *Plasmodium* species groups. In *P. vivax* and related simian *Plasmodium* species, the distances between A-type and S-type variants ranged between 7.8% and 10.9% (mean 9.5%) (Additional file 1). Distances between variants were considerably higher in *P. ovale curtisi* and *P. ovale wallikeri* with 17.0% and 17.6%, respectively. In the subgenus *Laverania*, distances between A-type and S-type clusters ranged from 11.4% to 12.9% (mean 12.1%), whereas in the rodent malaria subgenus *Vinckeia* they ranged from 8.0% to 11.3% (mean 9.9%). The RDP4 [58, 59] analyses detected recombinant signals in *18S* rDNA variants of all four *Plasmodium* species belonging to the subgenus *Laverania*, two species belonging to the subgenus *Vinckeia* (*P. berghei* and *P. yoelii*), and five species belonging to the subgenus *Plasmodium* (*P. coatneyi*, *P. cynomolgi*, *P. knowlesi*, *P. vivax*, and *P. ovale curtisi*) (Additional file 7). The four *Laverania* species featured the lowest GC-contents ranging from 32.3% to 35.7% (mean 33.9%). The values were higher in the four species of the subgenus *Vinckeia* with 35.9% to 38.8% (mean 37.4%). The GC-contents of the species group related to *P. vivax* ranged from 34.7% to 40.2% (mean 37.3%), whereby the O-type variant of *P. vivax* showed the highest value. The two *P. ovale* subspecies featured *18S* rDNAs with GC-contents from 36.0% to 37.5% (mean 36.6%), and *P. malariae* showed GC-contents ranging from 33.2 to 34.2 (mean 33.8%). The approximate total lengths of the *18S* rDNAs ranged from 2039 to 2676 bp (mean 2121 bp). The longest sequences were those of the S-type variants of *P. ovale wallikeri* and *P. ovale curtisi* with 2559 bp and 2676 bp, respectively, followed by the *P. vivax* O-type variant with 2216 bp. The *P. malaria* genome (PRJEB14392) features a unique 3350 bp long *18S* rDNA copy on chromosome 11, which was not included in the alignment because it aligned only partially with the other *Plasmodium* sequences. It remains unclear whether this aberrant variant is functional, because *18S* rDNA expression patterns have not been studied yet in *P. malariae*.

### *18S* rDNAs of *Haemoproteus* species

**Fig. 3:**
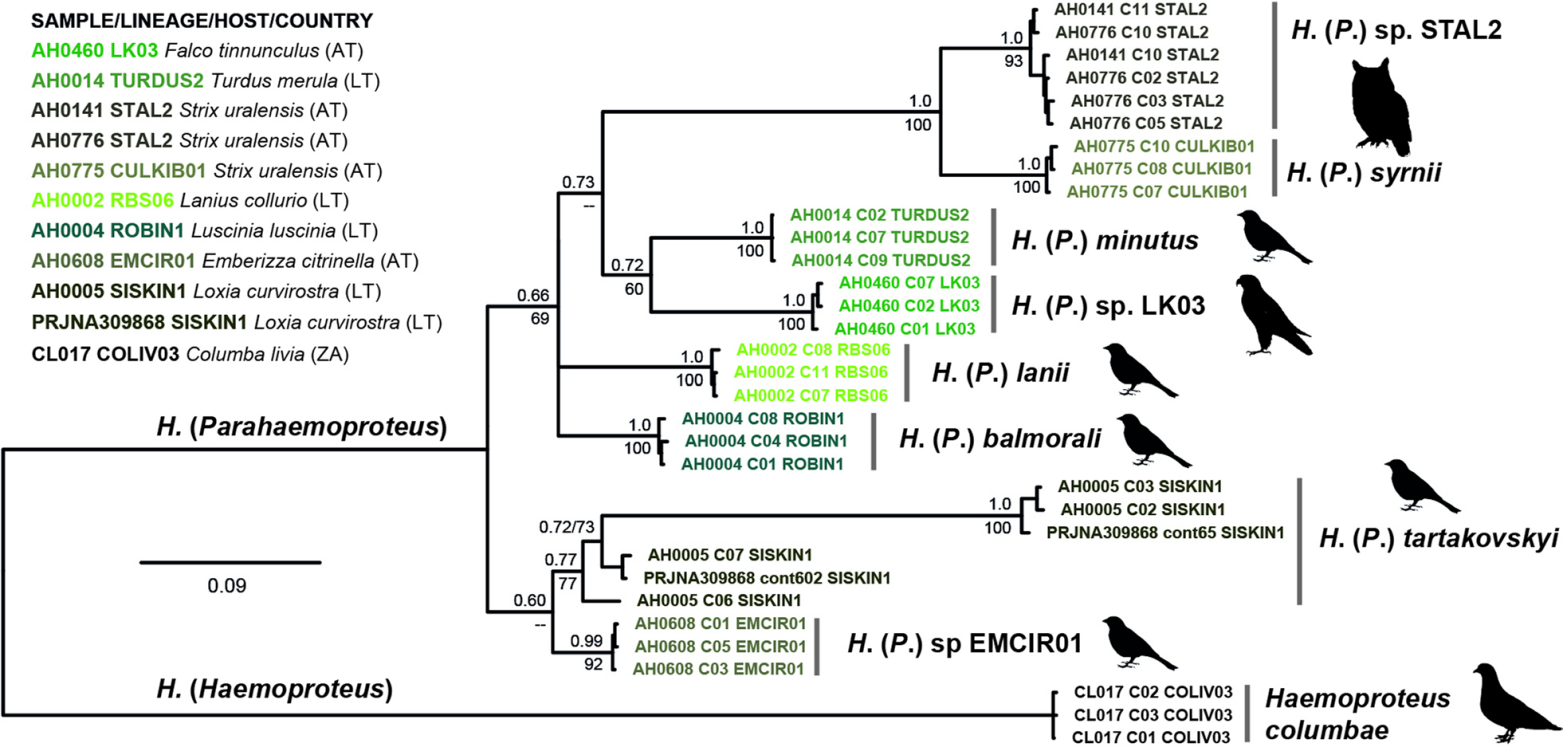
Bayesian inference tree of *Haemoproteus 18S* rDNA sequences. Posterior probabilities and maximum likelihood bootstrap values are indicated at most nodes. The scale bar indicates the expected mean number of substitutions per site according to the model of sequence evolution applied. The tree was midpoint-rooted, no outgroup was used

The *Haemoproteus* data set comprised sequences of eight species of the subgenus *Parahaemoproteus* and one sample of the subgenus *Haemoproteus*, *Haemoproteus columbae* (92 clones, 9.2 mean per sample). For taxonomic reasons, the *Parahaemoproteus* trees were rooted with *H. columbae*, although the *18S* rDNAs differed strongly between parasites of the two subgenera. The *Parahaemoproteus 18S* rDNAs differed from those of other haemosporidians (including *H. columbae*) by 31% to 32% and featured clearly distinct sequence patterns. Most *Parahaemoproteus* species clades were well supported in the *18S* rDNA trees, except for the *Haemoproteus* (*P.*) *tartakovskyi* clade (0.7/70), but relationships between species-clades were mostly not resolved (Fig. 3). The *18S* rDNA variants of the *Parahaemoproteus* parasites were similar within, but distinct between most species. The *p*-distances between clones were low in *Haemoproteus* (*P.*) *minutus* (AH0014H), *Haemoproteus* (*P.*) *lanii* (AH0002H), *Haemoproteus* (*P.*) sp. (AH0608H), and *Haemoproteus* (*P.*) *balmorali* (AH0004H) with up to 0.1%, 0.4%, 0.6%, and 0.9%, respectively. The latter four samples featured several different haplotypes each, but these did not form distinct clusters with unique sequence patterns. The intraspecific *p*-distances were only slightly higher in *Haemoproteus* (*P.*) sp. LK03 (AH0640H), *Haemoproteus* (*P.*) sp. STAL2 (AH0141H, AH0776H), and *Haemoproteus* (*P.*) *syrnii* (AH0775H) with 1.0%, 1.2%, and 1.3%, respectively. Each of the latter taxa featured at least three variants, indicating the presence of several distinct genomic *18S* rDNA copies. The *Haemoproteus* (*P.*) *tartakovskyi* sample (AH0005H) was exceptional, because the clones grouped into three clearly distinct sequence clusters. The haplotypes of the first cluster (clones 1, 2, 3, 4, 5, 8 and 9) differed from that of the second (clones 7 and 11) and the third cluster (clone 6) by 21.5% and 18.7%, respectively. The sequences of the latter two clusters differed from each other by 6.3%. The RDP4 [58, 59] analyses detected recombinant signals in several clones belonging to *Haemoproteus* (*P.*) *tartakovskyi* (AH0005H), *Haemoproteus* (*P.*) *syrnii* (AH0775H), and *Haemoproteus* (*P.*) *s*p. STAL2 (AH0141H, AH0776H), whereby at least two of five tests showed significant results for all of the latter. For three clones (AH0005H_C06, AH0775_C02, and AH0775_C10), significant results were obtained in four of five recombination tests (Additional file 7). The GC-content of the *Parahaemoproteus 18S* rDNAs ranged from 37.4% to 47.4% (mean 44.9%), and the approximate total lengths ranged from 1939 to 2176 bp. The GC-content of the *Haemoproteus columbae* (CL017) *18S* rDNAs was lower (36.5%), the approximate total length of the *18S* rDNAs was greater with 2280 bp. The *Haemoproteus columbae* haplotypes differed only in 0.4% from each other and no subdivision into distinct variants was evident.

### *18S* rDNAs of *Leucocytozoon* species

**Fig. 4:**
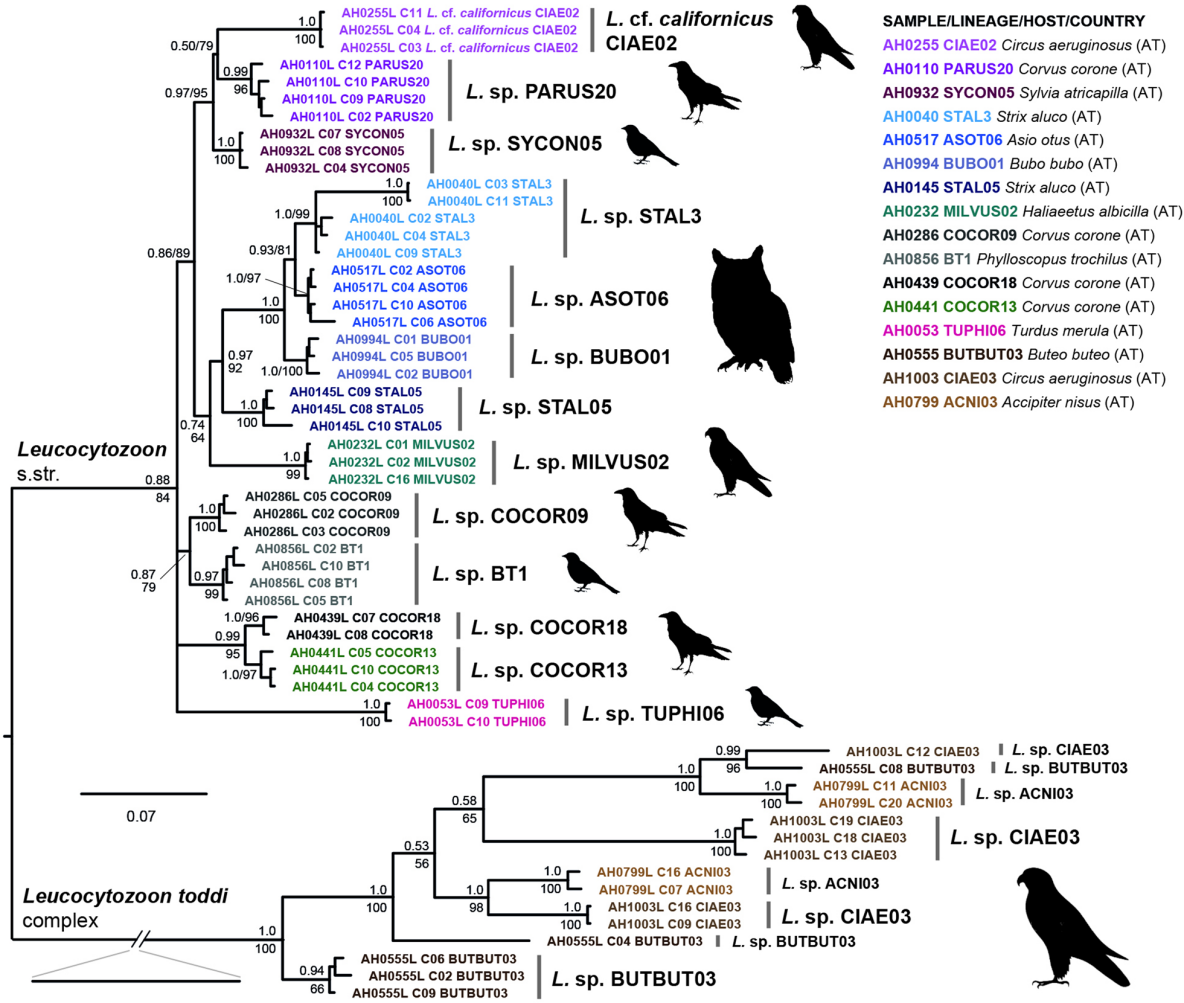
Bayesian inference tree of *Leucocytozoon 18S* rDNA sequences. Posterior probabilities and maximum likelihood bootstrap values are indicated at most nodes. The scale bar indicates the expected mean number of substitutions per site according to the model of sequence evolution applied. As outgroup a sequence of *P. relictum* (MalAvi lineage SGS1; AH0013P_c05) was included

The *Leucocytozoon* data set comprised *18S* rDNAs of 16 species (167 clones, 10.4 mean per sample). The trees were rooted with a sequence of *P. relictum* and featured to main clades, one clade with sequences of 13 *Leucocytozoon* species, and a second one with sequences of the three species belonging to the *Leucocytozoon toddi* species complex. The *18S* rDNAs of most *Leucocytozoon* species in the first clade were similar and grouped into reciprocally monophyletic clades (Fig. 4).

The lowest distances between intraspecific *18S* rDNA variants were found in *Leucocytozoon* sp. TUPHI06 (AH0053L), *Leucocytozoon* sp. SYCON5 (AH0932L), *Leucocytozoon* sp. MILVUS2 (AH0232L), and *Leucocytozoon* sp. COCOR18 (AH0439L) with up to 0.3%, 0.4%, 0.5%, 0.8%, respectively. The maximum distance between variants was only slightly higher in *Leucocytozoon* cf. *californicus* CIAE02 (AH0255L) and *Leucocytozoon* sp. BUBO01 (AH0994L) with 0.9% each. No structuring of haplotypes into different clusters was evident in the latter samples. The *18S* rDNAs of *Leucocytozoon* sp. COCOR09 (AH0286L), *Leucocytozoon* sp. BT1 (AH0856L), *Leucocytozoon* sp. PARUS20 (AH0110L), and *Leucocytozoon* sp. COCOR13 (AH0441L) differed by up to 1.3%, 1.6%, 1.8%, and 2.0%, respectively. The first two samples featured at least three, the latter two four distinct variants. The distances were slightly higher in *Leucocytozoon* sp. STAL5 (AH0145L), *Leucocytozoon* sp. ASOTO6 (AH0517L) and *Leucocytozoon* sp. STAL3 (AH0040L) with up to 2.2%, 2.3% and 5.7%, respectively. The first two species featured at least four distinct variants. Clone 6 of *Leucocytozoon* sp. ASOTO6 (AH0517L) differed considerably from the others, but only in a short sequence section (bp 1400 to 1600). The clones of *Leucocytozoon* sp. STAL3 (AH0040L) formed two clusters, with three similar haplotypes in the first, and clones 3 and 11 in the second cluster, both of the latter featuring clearly distinct sequence variants. The *18S* rDNA GC-content of the 13 *Leucocytozoon* species ranged from 34.0 to 38.6% (mean 36.9%), and the approximate total lengths ranged from 2094 to 2138 bp.

The sequences of the three species belonging to the *Leucocytozoon toddi* species complex grouped into two or three distinct clusters each. The clusters did not form reciprocally monophyletic clades, but clusters of different species grouped. The *p*-distances between *18S* rDNA variants of *Leucocytozoon* sp. BUTBUT03 (AH0555L), *Leucocytozoon* sp. ACNI03 (AH0799L) and *Leucocytozoon* sp. CIAE03 (AH1003L) were high with up to 16.9%, 17.4%, and 18.5%, respectively. One sequence cluster of *Leucocytozoon* sp. BUTBUT03 contained several distinct haplotypes with chimeric features (some haplotypes only partially matched two other clones, respectively), whereas the other two clusters contained only sequences of clones 4 and 8, respectively. Three sequence clusters were also identified in *Leucocytozoon* sp. CIAE03, two clusters of which together contained four distinct variants, and a third cluster which was represented by clone 12 only. The sequences of *Leucocytozoon* sp. ACNI03 formed two clusters, each containing at least three distinct variants. The RDP4 [58, 59] analyses detected recombinant signals in multiple *18S* clones of all three species belonging to the *Leucocytozoon toddi* complex. For some of the clones (AH0555L_C10, AH0555L_C12, AH0799_C16, AH1003L_C01, and AH1003L_C02) significant results were obtained in at least four of five recombination tests (Additional file 7). The species of the *Leucocytozoon toddi* species complex featured *18S* rDNAs with exceptionally high GC-contents, ranging between 47.0 and 50.5% (mean 49.3%). The approximate total lengths of the *18S* rDNAs ranged from 2125 to 2308 bp.

## Discussion

In the present study, the *18S* rDNAs of seven *Plasmodium*, nine *Haemoproteus*, and 16 *Leucocytozoon* species were analysed. Ten clones per lineage were sequenced in mean, whereby multiple samples were analysed of most avian *Plasmodium* species. The data might be more complete for the latter taxa, but it is possible that not all *18S* rDNA variants of the taxa investigated were obtained. The actual number of distinct *18S* rDNA variants could be determined in future studies by mapping rDNAs by restriction enzyme analysis and Southern blot hybridization as it was done in earlier studies on *P. berghei* [11], *P. falciparum* [16], and *P. cynomolgi* [19]. Another approach would be high-coverage sequencing, assembling the whole genomes of the parasites, and identifying the number and location of ribosomal units on the respective chromosomes. Therefore, further studies are required to determine the total number of ribosomal units in the species studied, and correctly assign them to specific chromosomes. In that context, the data cannot be used to formally test for a birth-and-death model.

Phylogenetic analyses were performed particularly to show the diversity of *18S* rDNA variants of the species studied, rather than aiming to resolve the relationships between those taxa. Generally, multi-copy genes, especially those of *Plasmodium* species, are not a good choice as phylogenetic markers for studying relationships between species. The human, simian and rodent *Plasmodium* species studied so far feature distinct ribosomal clusters, being differentially expressed in the vertebrate and mosquito hosts, and therefore subjected to different selective pressure [65]. Moreover, gene conversion between non-homologous genes also may generate misleading results [19]. Haemosporidian parasites feature the most variable nc ribosomal genes of all eukaryotes analysed to date, showing by far the highest intraspecific variability. Extremely variable sections cannot be aligned with confidence between distantly related taxa because of the presence of numerous insertion/deletions, even if secondary structure models are applied. Thus, more recent phylogenetic approaches for human, simian and rodent *Plasmodium* species rather tended to use the mt *CytB* [65] or nc coding genes [e.g., 66], whereas nc ribosomal genes were almost entirely neglected in avian haemosporidian research.

Although the species number of avian haemosporidians exceeds by far those in mammalian hosts, *18S* rDNAs have been published only from four avian *Plasmodium* species and *Haemoproteus* (*P.*) *tartakovskyi* [39]. Only the *18S* rDNA variants expressed in the vertebrate host (A-type) were sequenced from *P. lophurae* (X13706, [29]) and *P. cathemerium* (AY625607, [51]), and the *18S* rDNAs of the *P. relictum* genome (PRJEB9074; WTSI, unpublished) are fragmentary. The present data show that the patterns with several distinct *18S* rDNA variants (or ribosomal units) are not unique to human, simian and rodent malaria parasites, but are similar in most of the avian *Plasmodium* species studied belonging to three different subgenera. *Plasmodium* (*Haemamoeba*) *relictum*, *P.* (*Haemamoeba*) *gallinaceum*, *P.* (*Giovannolaia*) *homocircumflexum*, and *P.* (*Novyella*) *vaughani* each featured two sequence clusters, whereby the two clusters of each species grouped together in well-supported reciprocally monophyletic clades, suggesting that new variants originated independently in malaria parasites of different subgenera in the course of their evolution or that of related ancestral species. Differently, the two sequence clusters of *P.* (*Huffia*) *elongatum* and *P.* (*Haemamoeba*) *matutinum* were extremely diverged and did not group into reciprocally monophyletic clades, suggesting that they originated before the two species emerged. With exception of *P.* (*Novyella*) *delichoni* and *P. gallinaceum*, all *Plasmodium* species under investigation featured at least four clearly distinct sequence variants, indicating the presence of four or more genomic rDNA units. Apart from the *Plasmodium* species, the three species of the *Leucocytozoon toddi* complex also featured clearly distinct *18S* rDNA variants showing up in two or three clusters. Distinct variants of the latter species did not group in reciprocally monophyletic clades, but with those of the other species, suggesting that the last recent common ancestor of the *L. toddi* species complex already featured a set of different *18S* rDNA variants. Since only about ten clones per sample were sequenced and intraspecific diversity of haplotypes was extremely high, probably not all *18S* rDNA variants in the species studied were recovered. This species group was also peculiar regarding the high GC-content of their *18S* rDNAs (47.0% to 50.5%), exceeding that of *Parahaemoproteus* (37.4% to 47.4%), the other *Leucocytozoon* species (34.9% to 38.6%), *Haemoproteus* (36.5%), and *Plasmodium* (33.2% to 38.8%).

The patterns were different in the other *Leucocytozoon* species, with most species featuring similar haplotypes, which grouped in reciprocally monophyletic clades. Most *Parahaemoproteus* species and *Haemoproteus columbae* contained sets of similar clones, only *Haemoproteus* (*P.*) *tartakovskyi* featured three clearly distinct sequence variants, which grouped into a reciprocally monophyletic clade (Fig. 3). It cannot be excluded that the other seven *Parahaemoproteus* species contained additional, undetected *18S* rDNA copies because the primers were designed based on sequences of the *Haemoproteus* (*P.*) *tartakovskyi* genome only. However, it seems unlikely that the primers would not have recovered additional variants in at least some of the other seven species.

Expression of *18S* rDNA genes has not been studied in avian haemosporidians yet (except for that of the A-type variants in *P. lophurae*; [25]), but the present data indicate that differential expression may also occur in avian *Plasmodium* species and some other haemosporidians. The simian and rodent *Plasmodium* species each featured at least two distinct *18S* rDNAs clusters, the less diverged (short-branched) clusters contain the A-type variants expressed in the vertebrate host, and the more diverged (long-branched) clusters contain the S-type variants expressed in the mosquito hosts. A similar pattern is also evident in most avian *Plasmodium* species studied, because these also featured short-branched and long-branched clusters each (Fig. 2). However, additional experiments are required to clarify whether *18S* rDNAs of avian *Plasmodium* species (and species of related haemosporidian genera) show similar expression patterns as the human, simian and rodent *Plasmodium* species. The new data may provide the basis for studying *18S* rDNA expression of different developmental stages in the avian *Plasmodium* species by targeting rRNAs of distinct clusters with specific oligonucleotide probes in both the bird and mosquito hosts. Molecular hybridization assays could also be performed on the species of the *Leucocytozoon toddi* species complex and *Haemoproteus* (*P.*) *tartakovskyi*, to study if their rDNA units are expressed differentially as well. Due to the high number of considerably distinct *18S* rDNA clusters, these species would be promising candidates to test stage-specific expression of distinct ribosomal units in haemosporidian parasites belonging to genera other than *Plasmodium*.

The results of the recombination tests indicate that at least some species in all three genera studied have *18S* rDNA variants with chimeric features to a varying extent, potentially resulting from recombination events. This finding might relate to the general evolution and maintenance of ribosomal units in apicomplexan parasites. Whereas ribosomal units of most eukaryotes are assumed to evolve in a concerted fashion involving mechanisms such as unequal crossing-over during recombination, gene duplication, and inter-chromosomal gene conversion [6], the evolution of ribosomal units in malaria parasites was explained with a birth-and-death model, assuming that new variants originate by gene duplication and others are inactivated or deleted from the genome [9, 67]. However, the evolution of ribosomal units in apicomplexans is probably more complicated and involves mechanisms other than gene duplication and deletion only. Corredor & Enea [19] analysed the *18S* rDNA sequence patterns of *P. cynomolgi*, *P. berghei*, and *P. falciparum* and concluded that the two main gene types do not evolve independently but interact through gene conversion or some form of recombination [19, 68]. They found that the extent of divergence between sequence regions of isomorphs varies erratically and that the same regions show varying degrees of divergence depending on the species analysed. In this context, they point out that the ribosomal RNAs of *Plasmodium* species rather evolve in a semi-concerted manner [19]. The present data generally support this assumption, and the recombination tests detected chimeric features not only in *18S* rDNA alignments of human, simian and rodent *Plasmodium* species, but also in several avian *Plasmodium*, *Leucocytozoon*, and *Haemoproteus* species (Additional file 7). The finding that recombinant features were not detected in all species investigated, does not necessarily mean that their ribosomal genes were not affected by recombination, because not all *18S* rDNA variants might have been recovered (particularly in the genus *Haemoproteus*), or recombinant variants might have been deleted throughout evolution, thus obscuring past events. A general problem in avian haemosporidian research is that assembled and annotated genomes of *Leucocytozoon* and *Haemoproteus* parasites are still missing, and neither has the number and location of distinct ribosomal units been determined in any species by mapping rDNA by restriction enzyme analysis and Southern blot hybridization like in the first studies on *18S* rDNAs of rodent, human, and simian *Plasmodium* species (e.g., [11, 16]). Nonetheless, the fundamental reasons for the maintenance of sequentially distinct ribosomal genes in haemosporidian parasites generally remain insufficiently investigated. Many non-haemosporidian parasites of the Apicomplexa (e.g., *Theileria* spp., *Babesia* spp., and *Hepatozoon* spp.) also show complex life cycles with arthropod vectors and vertebrate hosts, but they do not feature considerably distinct ribosomal variants.

Malaria parasites and related haemosporidians cause diseases in many bird species. Recent molecular studies show that widespread *Haemoproteus* parasites, which have been formerly considered to be relatively benign [69], are actually virulent and cause severe pathology and even mortality in non-adapted birds due to damage of organs by tissue stages [70]. However, diagnostics of these parasites remain insufficiently developed in avian hosts during tissue stage of development. Ribosomal genes constitute the most frequent RNA molecules in cells and therefore are the most common targets in hybridization assays targeting parasites in host tissue. In situ hybridization techniques are particularly important in pathological studies, because they allow studying the effects of parasite infections in host tissues before the development of parasitemia. Additionally, this sensitive approach provides opportunities to distinguish development stages of haemosporidians belonging to different genera even during co-infections, which predominate in wildlife. The new data allow developing oligonucleotide probes specifically targeting and differentiating genera or species in host tissue. A chromogenic in situ hybridization protocol targeting the *18S* rRNAs of *Plasmodium* species in paraffin wax-embedded tissue samples of penguins was already developed [71]. The protocol was successfully applied to a wider range of parasite species [72, 73] and should allow for identification of all avian *Plasmodium* species belonging to different subgenera [personal comment based on the sequence data of the present study, JH]. Based on the *18S* rDNA data of the present study, Himmel et al. [48] developed chromogenic in situ hybridization protocols, which reliably allowed detecting blood and tissue stages of parasites belonging to *Haemoproteus* (*Parahaemoproteus*) species and *Leucocytozoon* spp. in paraffin wax-embedded tissue samples of birds by targeting the parasites’ ribosomes with genus specific probes.

In human malaria research, *18S* rDNAs are also the most common targets in PCR screening approaches. The first comprehensive PCR assay with species-specific primers targeting the *18S* rDNAs of the four human malaria species *P. falciparum*, *P. malariae*, *P. vivax* and *P. ovale* was published by [41]. Several other PCR protocols for the detection of simian *Plasmodium* species were developed later [43–45]. Differently, PCR assays developed for avian haemosporidians almost exclusively target mitochondrial genes [47, 74, 75]. In contrast to mitochondrial coding genes, the *18S* rDNAs feature multiple sequence sections being entirely conserved within haemosporidian parasites of certain genera but differing considerably between species of different genera. Based on the new *18S* rDNA data, PCR screening assays could be developed to reliably detect and differentiate between avian haemosporidians belonging to different genera.

## Conclusion

In the present study, *18S* rDNA sequences of 32 avian haemosporidian species were sequenced, which more than doubles the number of haemosporidian taxa for which *18S* sequences are available. Moreover, literature on *18S* rDNAs of human, simian and rodent *Plasmodium* species was reviewed, and already published data was included in the analyses to compare patterns with those found in the avian haemosporidians. The present data show that gene duplication events giving rise to two diverged main sequence clusters happened independently in at least six out of seven avian *Plasmodium* species, thus supporting evolution according to a birth-and-death model like proposed for the ribosomal units of human, simian and rodent *Plasmodium* species. The *18S* rDNA patterns of the three species of the *Leucocytozoon toddi* complex and *Haemoproteus tartakovskyi* also support evolution according to the latter model. However, the other species featured sets of similar *18S* rDNA haplotypes, raising the question of whether their ribosomal units rather evolve in concerted fashion like in other eukaryotes (including most non-haemosporidian apicomplexans). Moreover, the presence of chimeric *18S* rDNA variants in some *Plasmodium* and *Leucocytozoon* species indicates that the distinct ribosomal units are not evolving entirely independently, but rather in a semi-concerted evolution as suggested by [19]. The new data may facilitate studying differential expression of distinct ribosomal units in haemosporidians other than human, simian and rodent *Plasmodium* species. In a study published recently [48], using the *18S* rDNA data of the present study, hybridization assays with genus-specific probes for *Leucocytozoon* and *Haemoproteus* (subgenus *Parahaemoproteus*), targeting the parasites’ ribosomes in host tissue, were already developed.

## Supplementary information

**Additional file 1.** Human, simian and rodent malaria species included in the *Plasmodium* dataset.

**Additional file 2.** Bayesian inference trees calculated from *CytB* sequences of *Plasmodium* species (A), *Haemoproteus* spp. (B) and *Leucocytozoon* spp. (C). The trees were midpoint-rooted, no outgroups were used. Posterior probabilities and maximum likelihood bootstrap values are indicated at most nodes. The scale bar indicates the expected mean number of substitutions per site according to the model of sequence evolution applied.

**Additional file 3.** Maximum-likelihood tree of *Plasmodium 18S* rDNA sequences based on a secondary structure alignment calculated with R-Coffee and only gaps trimmed.

**Additional file 4.** Maximum-likelihood tree of *Plasmodium 18S* rDNA sequences based on a secondary structure alignment calculated with R-Coffee applying the “strict” algorithm implemented in trimAl v.1.2 [53] for trimming.

**Additional file 5.** Maximum-likelihood tree of *Plasmodium 18S* rDNA sequences based on a secondary structure alignment calculated with R-Coffee applying the default option on the G-blocks Server [55] for trimming.

**Additional file 6.** Sequence features of the *18S* rDNAs analysed.

**Additional file 7.** Results of the recombination tests.

## Data Availability

All DNA, blood and tissue samples are deposited in the collection of the Institute of Pathology (Department of Pathobiology, University of Veterinary Medicine Vienna). All sequence data were uploaded to NCBI GenBank. DNA sequence alignments are available from J.H. upon request.

## Abbreviations

nc: nuclear
mt: mitochondrial
rDNA: ribosomal DNA
rRNA: ribosomal RNA
*Cytb*: cytochrome b
SSU: small ribosomal subunit
LSU: large ribosomal subunit
NTS: non-transcribed spacer
ETS: external transcribed spacer
ITS: internal transcribed spacer
MHC: major histocompatibility complex
