# Supplementary material for: The nuclear 18S ribosomal DNAs of avian haemosporidian parasites
Source: Malar J. 2019 Sep 3;18:305. doi: 10.1186/s12936-019-2940-6 (PMC6724295; doi:10.1186/s12936-019-2940-6)
Supplement: Supplementary file 1 — Additional file 1. Human, simian and rodent malaria species included in the Plasmodium dataset. [file 12936_2019_2940_MOESM1_ESM.docx]

**Additional file S1:** Human, simian and rodent malaria species included in the *Plasmodium* dataset.

| **Accession/Project IDs** | **Host group** | **Species** | **Clones/Chromosomes/Contigs** | **18S mean length (bp)** | **GC content mean (%)** | **Max. *p*-dist. (%)** |
| --- | --- | --- | --- | --- | --- | --- |
| PRJNA20431 | human | *Plasmodium* (*Plasmodium*) *vivax* | ch2, ch5, ch6 | 2148 | 0,38 | 8.9/17.2 |
| PRJNA315987 | simian | *Plasmodium* (*Plasmodium*) *coatneyi* | ch3, ch5, ch10 | 2085 | 0,38 | 9.4 |
| AB287288–90 | simian | *Plasmodium* (*Plasmodium*) *cynomolgi* | cl1, cl4, cl7 | 2163 | 0,38 | 10.9 |
| AB287281–83 | simian | *Plasmodium* (*Plasmodium*) *fieldi* | cl22, cl23, cl27 | 2169 | 0,38 | 9.0 |
| AB287272–74 | simian | *Plasmodium* (*Plasmodium*) *fragile* | cl55, cl58, cl61 | 2183 | 0,36 | 9.8 |
| AB287269–71 | simian | *Plasmodium* (*Plasmodium*) *gonderi* | cl7, cl9, cl16 | 2096 | 0,36 | 7.8 |
| AB287278–80 | simian | *Plasmodium* (*Plasmodium*) *hylobati* | cl7, cl11, cl25 | 2168 | 0,38 | 10.0 |
| AB287275–77 | simian | *Plasmodium* (*Plasmodium*) *inui* | cl12, cl109, cl120 | 2171 | 0,38 | 10.3 |
| AB287285–87 | simian | *Plasmodium* (*Plasmodium*) *simiovale* | cl55, cl56, cl57 | 2165 | 0,38 | 10.2 |
| PRJEB19298 | simian/human | *Plasmodium* (*Plasmodium*) *knowlesi* | ch3, ch10, ch13 | 2158 | 0,37 | 8.5 |
| PRJEB12678 | human | *Plasmodium* (*Plasmodium*) *ovale curtisi* | cont125, cont2140, cont2646 | 2102 | 0,36 | 17.0 |
| PRJEB12679 | human | *Plasmodium* (*Plasmodium*) *ovale wallikeri* | cont5, cont505, cont910 | 2093 | 0,36 | 17.6 |
| PRJEB14392 | human | *Plasmodium* (*Plasmodium*) *malariae* | ch3, ch10 | 2157 | 0,34 | 0.3 |
| PRJNA13173 | human | *Plasmodium* (*Laverania*) *falciparum* | ch1, ch5, ch7, ch11, ch13 | 2150 | 0,33 | 12.3 |
| PRJEB13584 | simian | *Plasmodium* (*Laverania*) *gaboni* | ch1, ch5, ch7, ch13 | 2081 | 0,36 | 12.9 |
| PRJEB13584 | simian | *Plasmodium* (*Laverania*) sp. (gorilla G3) | ch1, ch5, ch7, ch13 | 2100 | 0,35 | 11.4 |
| PRJEB13584 | simian | *Plasmodium* (*Laverania*) *reichenowi* | ch1, ch5, ch7, ch11 | 2094 | 0,36 | 11.8 |
| PRJNA317456 | rodent | *Plasmodium* (*Vinckeia*) *berghei* | ch5, ch6, ch7 | 2064 | 0,37 | 8.0 |
| PRJNA317457 | rodent | *Plasmodium* (*Vinckeia*) *chabaudi* | ch5, ch9 | 2062 | 0,37 | 11.3 |
| PRJNA255250 | rodent | *Plasmodium* (*Vinckeia*) *vinckei vinckei* | con165, cont172 | 2063 | 0,37 | 10.5 |
| PRJNA163125 | rodent | *Plasmodium* (*Vinckeia*) *vinckei petteri* | cont168, cont187 | 2062 | 0,37 | 10.7 |
| PRJNA317465 | rodent | *Plasmodium* (*Vinckeia*) *yoelii* | ch5, ch6, ch7, ch12 | 2064 | 0,36 | 9.2 |

Mean GC-content (in percent), approximate total lengths, and maximum *p*-distances between *18S* rDNA variants.
