# Supplementary figures and images for: The nuclear 18S ribosomal DNAs of avian haemosporidian parasites
Source: Malar J. 2019 Sep 3;18:305. doi: 10.1186/s12936-019-2940-6 (PMC6724295; doi:10.1186/s12936-019-2940-6)

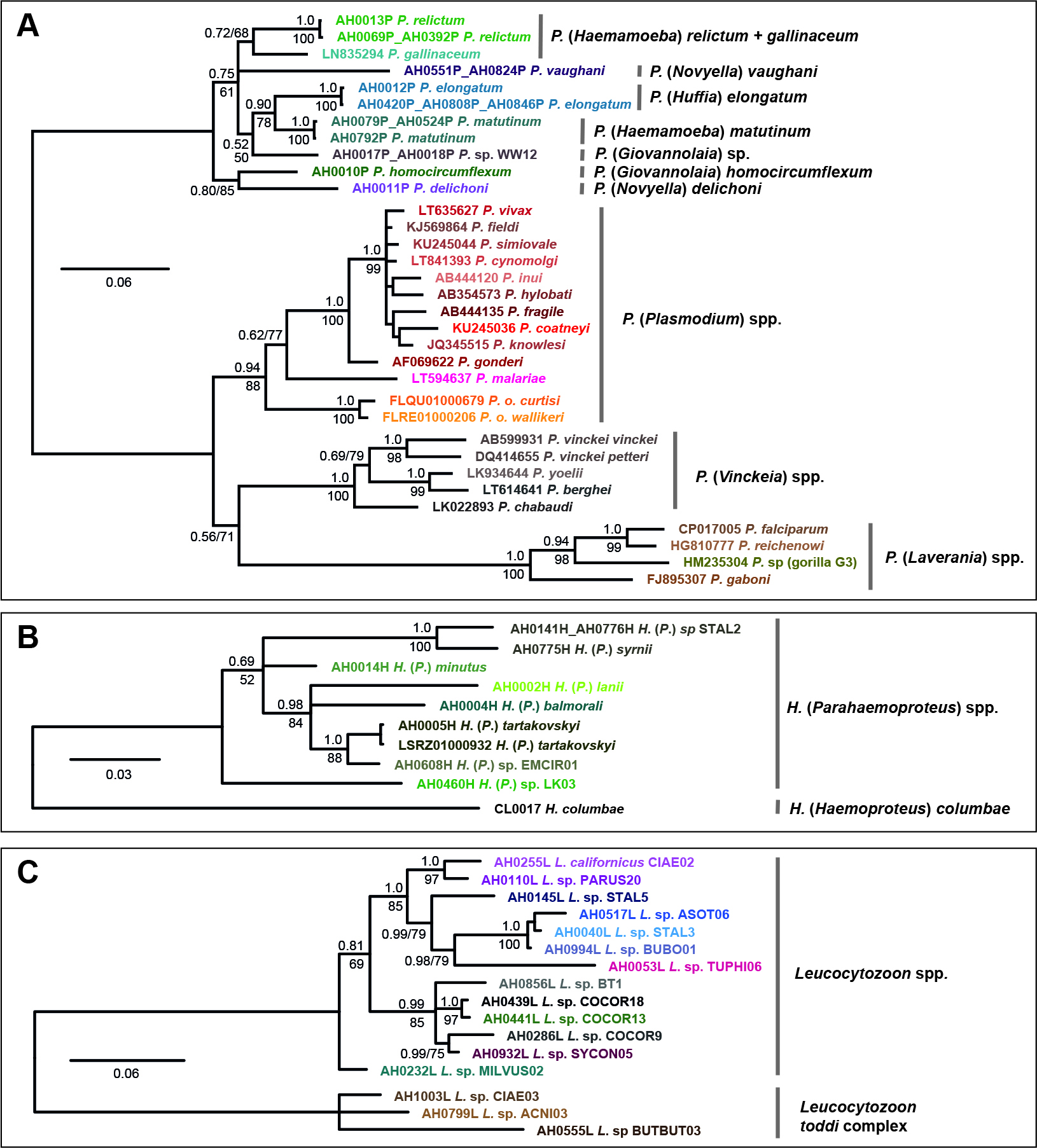

Supplement: Supplementary file 2 — Additional file 2. Bayesian inference trees calculated from CytB sequences of Plasmodium species (A), Haemoproteus spp. (B) and Leucocytozoon spp. (C). The trees were midpoint-rooted, no outgroups were used. Posterior probabilities and maximum likelihood bootstrap values are indicated at most nodes. The scale bar indicates the expected mean number of substitutions per site according to the model of sequence evolution applied. [file 12936_2019_2940_MOESM2_ESM.jpg]

Alignment method: RCoffee (default)

Trimming: gaps excluded

final alignment: 1555 bp

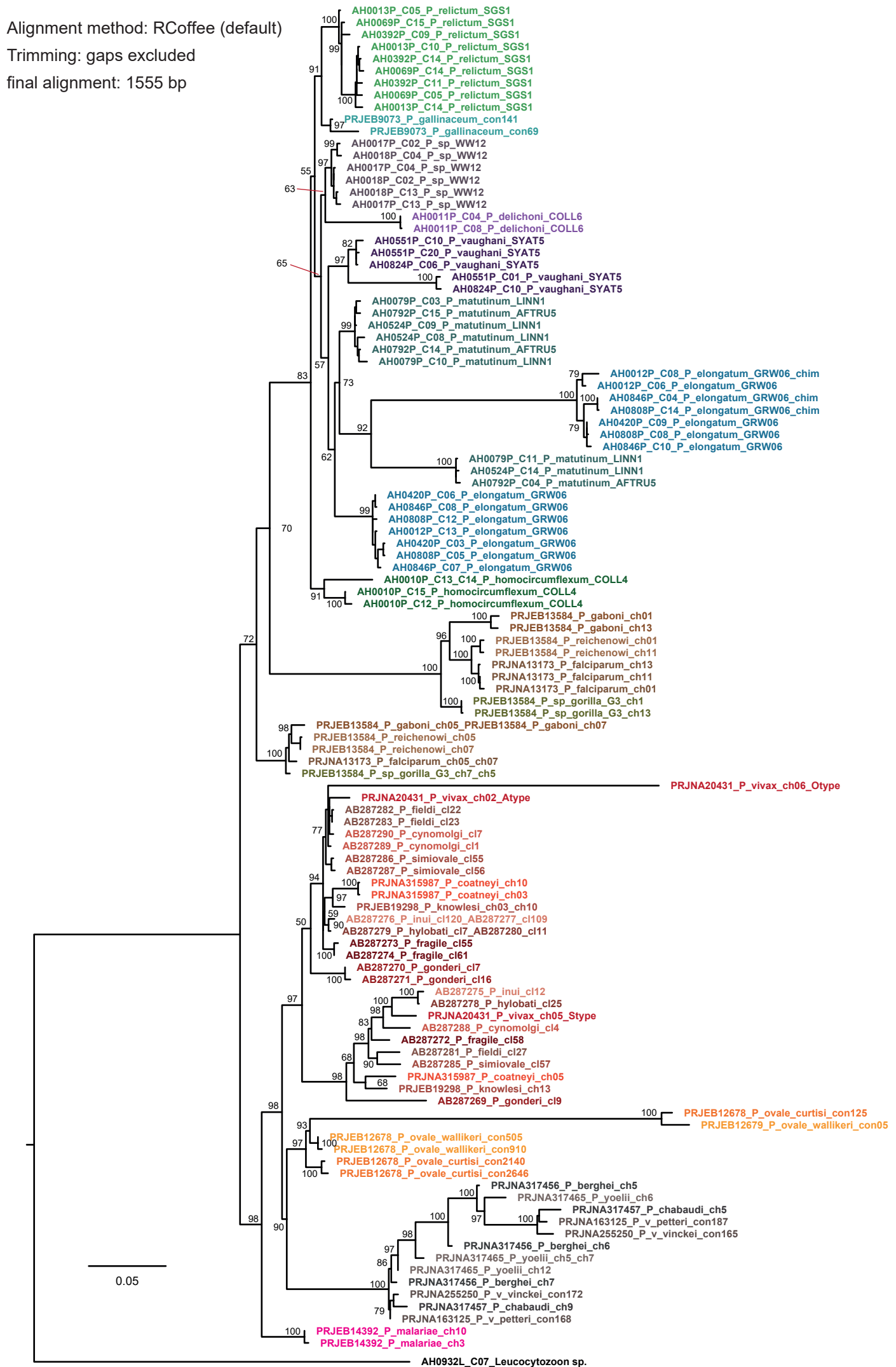

Supplement: Supplementary file 3 — Additional file 3. Maximum-likelihood tree of Plasmodium 18S rDNA sequences based on a secondary structure alignment calculated with R-Coffee and only gaps trimmed. [file 12936_2019_2940_MOESM3_ESM.pdf]

Alignment method: RCoffee (default)

Trimming: GBLOCK (default)

final alignment: 1360 bp

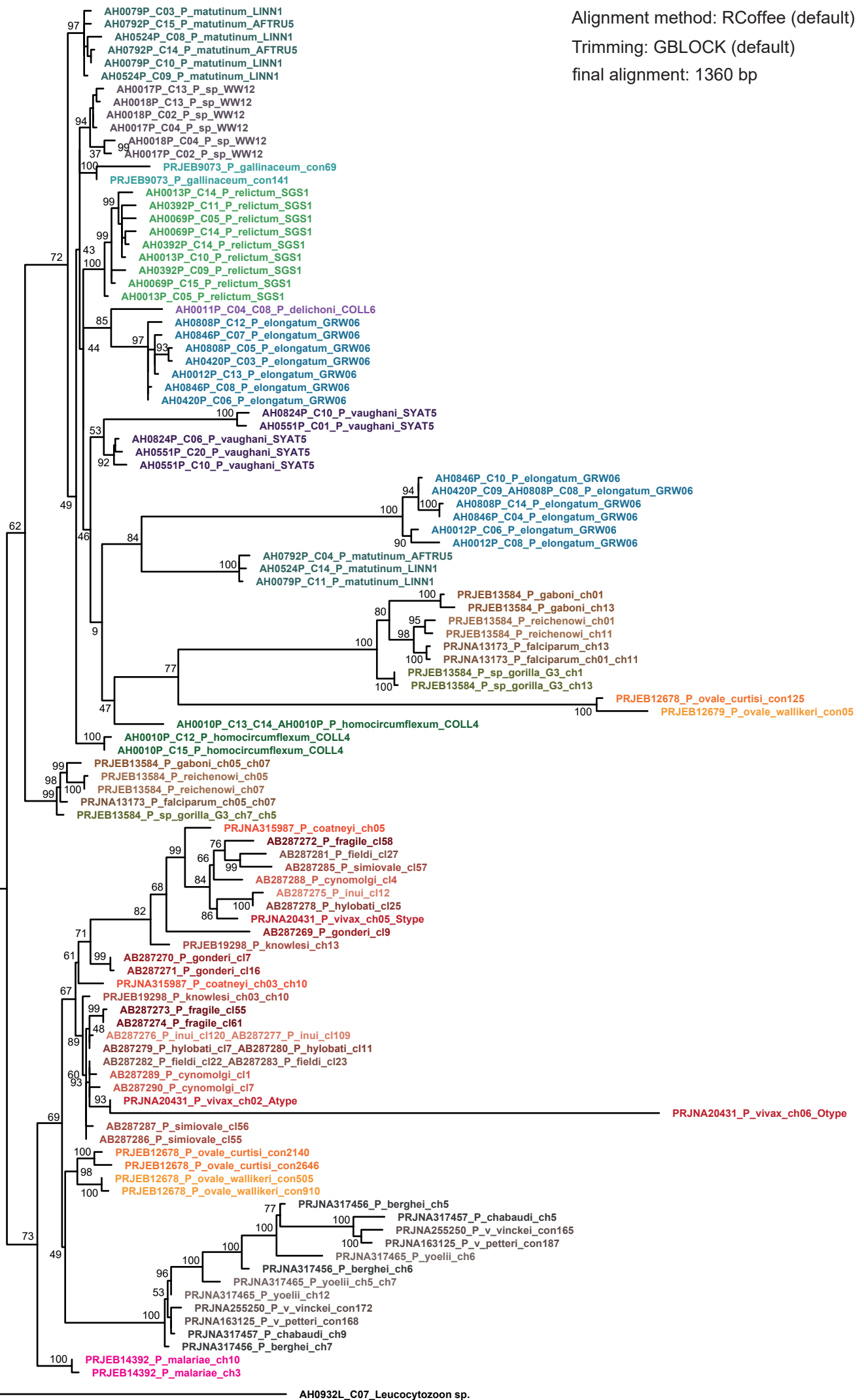

Supplement: Supplementary file 5 — Additional file 5. Maximum-likelihood tree of Plasmodium 18S rDNA sequences based on a secondary structure alignment calculated with R-Coffee applying the default option on the G-blocks Server [55] for trimming. [file 12936_2019_2940_MOESM5_ESM.pdf]
