# Supplementary material for: The nuclear 18S ribosomal DNAs of avian haemosporidian parasites
Source: Malar J. 2019 Sep 3;18:305. doi: 10.1186/s12936-019-2940-6 (PMC6724295; doi:10.1186/s12936-019-2940-6)
Supplement: Supplementary file 4 — Additional file 4. Maximum-likelihood tree of Plasmodium 18S rDNA sequences based on a secondary structure alignment calculated with R-Coffee applying the “strict” algorithm implemented in trimAl v.1.2 [53] for trimming. [file 12936_2019_2940_MOESM4_ESM.pdf]

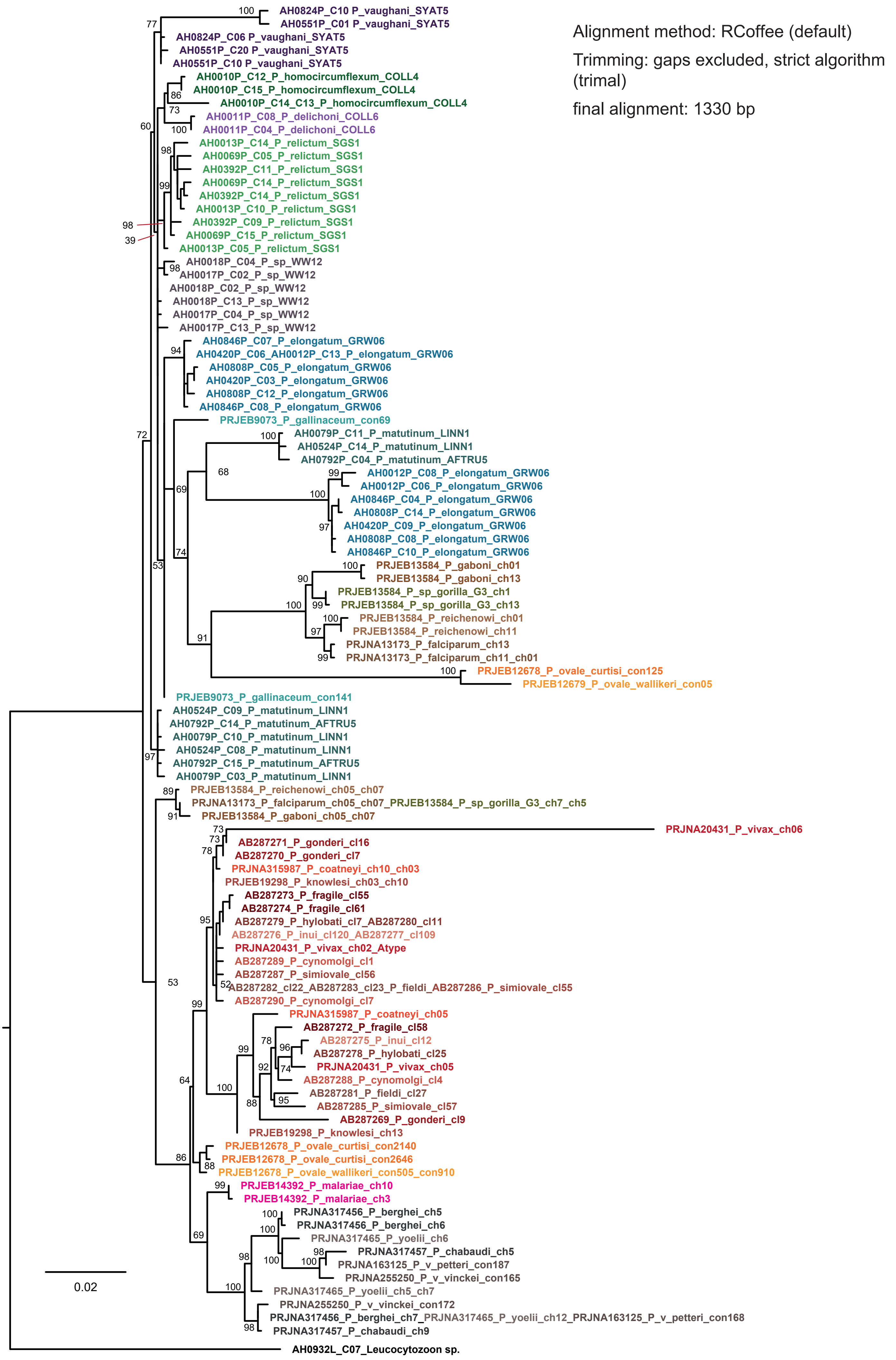

Alignment method: RCoffee (default)
Trimming: gaps excluded, strict algorithm (trimal)
final alignment: 1330 bp
AH0824P_C10 P_vaughani_SYAT5
AH0551P_C01 P_vaughani_SYAT5
AH0824P_C06 P_vaughani_SYAT5
AH0551P_C20 P_vaughani_SYAT5
AH0551P_C10 P_vaughani_SYAT5
AH0010P_C12_P_homocircumflexum_COLL4
AH0010P_C15_P_homocircumflexum_COLL4
AH0010P_C14_C13_P_homocircumflexum_COLL4
AH0011P_C08_P_delichoni_COLL6
AH0011P_C04_P_delichoni_COLL6
AH0013P_C14_P_relictum_SGS1
AH0069P_C05_P_relictum_SGS1
AH0392P_C11_P_relictum_SGS1
AH0069P_C14_P_relictum_SGS1
AH0392P_C14_P_relictum_SGS1
AH0013P_C10_P_relictum_SGS1
AH0392P_C09_P_relictum_SGS1
AH0069P_C15_P_relictum_SGS1
AH0013P_C05_P_relictum_SGS1
AH0018P_C04_P_sp_WW12
AH0017P_C02_P_sp_WW12
AH0018P_C02_P_sp_WW12
AH0018P_C13_P_sp_WW12
AH0017P_C04_P_sp_WW12
AH0017P_C13_P_sp_WW12
AH0846P_C07_P_elongatum_GRW06
AH0420P_C06_AH0012P_C13_P_elongatum_GRW06
AH0808P_C05_P_elongatum_GRW06
AH0420P_C03_P_elongatum_GRW06
AH0808P_C12_P_elongatum_GRW06
AH0846P_C08_P_elongatum_GRW06
PRJEB9073_P_gallinaceum_con69
AH0079P_C11_P_matutinum_LINN1
AH0524P_C14_P_matutinum_LINN1
AH0792P_C04_P_matutinum_AFTRU5
AH0012P_C08_P_elongatum_GRW06
AH0012P_C06_P_elongatum_GRW06
AH0846P_C04_P_elongatum_GRW06
AH0808P_C14_P_elongatum_GRW06
AH0420P_C09_P_elongatum_GRW06
AH0808P_C08_P_elongatum_GRW06
AH0846P_C10_P_elongatum_GRW06
PRJEB13584_P_gaboni_ch01
PRJEB13584_P_gaboni_ch13
PRJEB13584_P_sp_gorilla_G3_ch1
PRJEB13584_P_sp_gorilla_G3_ch13
PRJEB13584_P_reichenowi_ch01
PRJEB13584_P_reichenowi_ch11
PRJNA13173_P_falciparum_ch13
PRJNA13173_P_falciparum_ch11_ch01
PRJEB12678_P_ovale_curtisi_con125
PRJEB12679_P_ovale_wallikeri_con05
PRJEB9073_P_gallinaceum_con141
AH0524P_C09_P_matutinum_LINN1
AH0792P_C14_P_matutinum_AFTRU5
AH0079P_C10_P_matutinum_LINN1
AH0524P_C08_P_matutinum_LINN1
AH0792P_C15_P_matutinum_AFTRU5
AH0079P_C03_P_matutinum_LINN1
PRJEB13584_P_reichenowi_ch05_ch07
PRJNA13173_P_falciparum_ch05_ch07_PRJEB13584_P_sp_gorilla_G3_ch7_ch5
PRJEB13584_P_gaboni_ch05_ch07
PRJNA20431_P_vivax_ch06
AB287271_P_gonderi_cl16
AB287270_P_gonderi_cl7
PRJNA315987_P_coatneyi_ch10_ch03
PRJEB19298_P_knowlesi_ch03_ch10
AB287273_P_fragile_cl55
AB287274_P_fragile_cl61
AB287279_P_hylobati_cl7_AB287280_cl11
AB287276_P_inui_cl120_AB287277_cl109
PRJNA20431_P_vivax_ch02_Atype
AB287289_P_cynomolgi_cl1
AB287287_P_simiovale_cl56
AB287282_cl22_AB287283_cl23_P_fieldi_AB287286_P_simiovale_cl55
AB287290_P_cynomolgi_cl7
PRJNA315987_P_coatneyi_ch05
AB287272_P_fragile_cl58
AB287275_P_inui_cl12
AB287278_P_hylobati_cl25
PRJNA20431_P_vivax_ch05
AB287288_P_cynomolgi_cl4
AB287281_P_fieldi_cl27
AB287285_P_simiovale_cl57
AB287269_P_gonderi_cl9
PRJEB19298_P_knowlesi_ch13
PRJEB12678_P_ovale_curtisi_con2140
PRJEB12678_P_ovale_curtisi_con2646
PRJEB12678_P_ovale_wallikeri_con505_con910
PRJEB14392_P_malariae_ch10
PRJEB14392_P_malariae_ch3
PRJNA317456_P_berghei_ch5
PRJNA317456_P_berghei_ch6
PRJNA317465_P_yoelii_ch6
PRJNA317457_P_chabaudi_ch5
PRJNA163125_P_v_petteri_con187
PRJNA255250_P_v_vinckei_con165
PRJNA317465_P_yoelii_ch5_ch7
PRJNA255250_P_v_vinckei_con172
PRJNA317456_P_berghei_ch7_PRJNA317465_P_yoelii_ch12_PRJNA163125_P_v_petteri_con168
PRJNA317457_P_chabaudi_ch9
AH0932L_C07_Leucocytozoon sp.
0.02
