# Supplementary material for: The nuclear 18S ribosomal DNAs of avian haemosporidian parasites
Source: Malar J. 2019 Sep 3;18:305. doi: 10.1186/s12936-019-2940-6 (PMC6724295; doi:10.1186/s12936-019-2940-6)
Supplement: Supplementary file 6 — Additional file 6. Sequence features of the 18S rDNAs analysed. [file 12936_2019_2940_MOESM6_ESM.docx]

**Additional file S6:** Sequence features of the *18S* rDNAs analyzed.

| **ID** | **MalAvi lineage** | **Species** | **No. clones** | ***18S* rDNA lengths** | **GC content mean (%)** | **Max. dist. (%)** |
| --- | --- | --- | --- | --- | --- | --- |
| AH0013P | SGS1 | *Plasmodium* (*Haemamoeba*) *relictum* | 11 | 2124–2131 | 33,7 | 2.5 |
| AH0069P | SGS1 | *P.* (*Haemamoeba*) *relictum* | 11 | 2124–2134 | 33,6 | 2.5 |
| AH0392P | SGS1 | *P.* (*Haemamoeba*) *relictum* | 10 | 2125–2130 | 33,7 | 2.5 |
| AH0792P | AFTRU5 | *P.* (*Haemamoeba*) *matutinum* | 12 | 2106–2164 | 33,1 | 10.8 |
| AH0524P | LINN1 | *P.* (*Haemamoeba*) *matutinum* | 9 | 2110–2162 | 34,1 | 10.8 |
| AH0079P | LINN1 | *P.* (*Haemamoeba*) *matutinum* | 10 | 2111–2164 | 33,1 | 10.8 |
| AH0012P | ERIRUB01 | *P.* (*Huffia*) *elongatum* | 10 | 2103–2162 | 34,0 | 14.9 |
| AH0420P | GRW06 | *P.* (*Huffia*) *elongatum* | 8 | 2101–2140 | 34,3 | 14.9 |
| AH0808P | GRW06 | *P.* (*Huffia*) *elongatum* | 10 | 2100–2154 | 34,2 | 14.9 |
| AH0846P | GRW06 | *P.* (*Huffia*) *elongatum* | 9 | 2101–2154 | 34,7 | 14.9 |
| AH0011P | COLL6 | *P.* (*Novyella*) *delichoni* | 10 | 2116–2119 | 35,0 | 0.6 |
| AH0551P | SYAT05 | *P.* (*Novyella*) *vaughani* | 11 | 2127–2177 | 33,2 | 9.3 |
| AH0824P | SYAT05 | *P.* (*Novyella*) *vaughani* | 6 | 2126–2176 | 33,1 | 9.3 |
| AH0010P | COLL4 | *P.* (*Giovannolaia*) *homocircumflexum* | 12 | 2116–2140 | 35,0 | 7.2 |
| AH0017P | WW12 | *P.* (*Giovannolaia*) sp. | 11 | 2117–2120 | 34,3 | 1.7 |
| AH0018P | WW12 | *P.* (*Giovannolaia*) sp*.* | 9 | 2116–2121 | 34,1 | 1.7 |
| CL017 | COLIV03 | *Haemoproteus* (*Haemoproteus*) *columbae* | 11 | 2282–2282 | 36,5 | 0.4 |
| AH0004H | ROBIN1 | *H.* (*Parahaemoproteus*) *balmorali* | 10 | 2125–2126 | 45,0 | 0.8 |
| AH0002H | RBS06 | *H.* (*Parahaemoproteus*) *lanii* | 10 | 2181–2185 | 43,9 | 0.4 |
| AH0014H | TURDUS2 | *H.* (*Parahaemoproteus*) *minutus* | 8 | 1939–1939 | 45,1 | 0.1 |
| AH0775H | CULKIB01 | *H.* (*Parahaemoproteus*) *syrnii* | 9 | 2173–2178 | 45,5 | 1.4 |
| AH0005H | SISKIN1 | *H.* (*Parahaemoproteus*) *tartakovskyi* | 10 | 2042–2093 | 39,0 | 20.4 |
| AH0141H | STAL2 | *H.* (*Parahaemoproteus*) sp. | 8 | 2137–2143 | 47,4 | 1.3 |
| AH0776H | STAL2 | *H.* (*Parahaemoproteus*) sp. | 11 | 2137–2142 | 47,3 | 1.3 |
| AH0460H | LK03 | *H.* (*Parahaemoproteus*) sp. | 9 | 2003–2004 | 46,7 | 1.0 |
| AH0608H | EMCIR01 | *H.* (*Parahaemoproteus*) sp. | 6 | 2108–2109 | 44,4 | 0.6 |
| AH0255L | CIAE02 | *Leucocytozoon* cf. *californicus* | 12 | 2106–2106 | 38,3 | 0.9 |
| AH0040L | STAL3 | *Leucocytozoon* sp. | 10 | 2116–2133 | 37,5 | 5.4 |
| AH0053L | TUPHI06 | *Leucocytozoon* sp. | 2 | 2102–2171 | 37,5 | 0.2 |
| AH0110L | PARUS20 | *Leucocytozoon* sp. | 10 | 2102–2103 | 38,6 | 1.8 |
| AH0145L | STAL5 | *Leucocytozoon* sp. | 10 | 2127–2132 | 36,5 | 2.3 |
| AH0232L | MILVUS02 | *Leucocytozoon* sp. | 11 | 2179–2183 | 34,9 | 0.5 |
| AH0286L | COCOR09 | *Leucocytozoon* sp. | 10 | 2107–2111 | 36,7 | 1.3 |
| AH0439L | COCOR18 | *Leucocytozoon* sp. | 2 | 2095–2101 | 38,3 | 0.8 |
| AH0441L | COCOR13 | *Leucocytozoon* sp. | 11 | 2094–2096 | 36,5 | 1.0 |
| AH0517L | ASOT06 | *Leucocytozoon* sp. | 10 | 2103–2117 | 37,1 | 2.3 |
| AH0856L | BT1 | *Leucocytozoon* sp. | 11 | 2098–2098 | 37,6 | 1.6 |
| AH0932L | SYCON05 | *Leucocytozoon* sp. | 10 | 2108–2108 | 38,2 | 0.4 |
| AH0994L | BUBO01 | *Leucocytozoon* sp. | 11 | 2111–2111 | 37,7 | 0.9 |
| AH0555L | BUTBUT03 | *Leucocytozoon* sp. (*L. toddi* complex) | 11 | 2162–2276 | 47,7 | 17.0 |
| AH0799L | ACNI03 | *Leucocytozoon* sp. (*L. toddi* complex) | 10 | 2125–2308 | 51,5 | 17.4 |
| AH1003L | CIAE03 | *Leucocytozoon* sp. (*L. toddi* complex) | 12 | 2193–2306 | 49,6 | 18.4 |

Mean GC-content (in percent), approximate total lengths, and maximum *p*-distances between *18S* rDNA variants.
